# Analysis of the Effect of Autoclaving on the Flavor of Fresh Ginseng Using Electronic Nose, HS-GC-IMS, and HS-SPME-GC-MS

**DOI:** 10.3390/foods15183289

**Published:** 2026-09-17

**Authors:** Junshun Zhang, Yuqi Li, Juanjuan Luo, Suwan Lu, Qishun Zhu, Linlin Yin, Shoubao Yan

**Affiliations:** 1 School of Biological Engineering, Huainan Normal University, Huainan 232038, China; zhangjunshun@hnnu.edu.cn (J.Z.);; 2 College of Food Science and Technology, Nanjing Agricultural University, Nanjing 210095, China

**Keywords:** ginseng, autoclaving, flavor, HS-GC-IMS, HS-SPME-GC-MS

## Abstract

Ginseng is an important plant with both medicinal and edible values, which are widely used after thermal processing. To investigate the effects of autoclaving on the flavor of fresh ginseng, electronic nose (E-nose), headspace–gas chromatography–ion mobility spectrometry (HS-GC-IMS) and headspace–solid phase microextraction–gas chromatography–mass spectrometry (HS-SPME-GC-MS) were employed to characterize the volatile organic compounds (VOCs) in autoclaved ginseng (AG) and fresh ginseng (FG). The results of E-nose detection revealed significant differences in the volatile components between AG and FG, with AG containing more abundant short-chain alkanes such as methane, organic sulfides and aromatic components. A total of 81 VOCs, mainly aldehydes, alcohols and esters, were qualitatively identified by GC-IMS. GC-MS analysis identified 1103 VOCs, among which 181 volatile components showed a downward trend and 72 exhibited an upward trend after the autoclaving process. Notably, terpenoids, esters and ketones exhibited significant differences in composition and abundance between FG and AG. This study elucidated the effects of autoclaving on the volatile flavor profile of fresh ginseng and offered critical theoretical insights for flavor analysis and quality improvement in ginseng processing, as well as the rational development of ginseng functional foods.

## 1. Introduction

Ginseng (*Panax ginseng* C.A. Mey.) is an important plant with both medicinal and edible values and has a long history of consumption. It is widely cultivated in East Asia and North America and contains a wealth of bioactive components, including ginsenosides, polysaccharides, amino acids and trace elements [1]. Modern pharmacological studies have confirmed that ginseng exhibits diverse bioactivities, including anti-inflammatory, antioxidant [2], anti-obesity [3], anti-tumor, anti-diabetic, and immunomodulatory activities [4], making it a valuable raw material for functional food development.

Nevertheless, fresh ginseng contains various hydrolases that readily hydrolyze and inactivate ginsenosides, and it is also highly susceptible to moisture-induced spoilage and mold growth [5]. Therefore, fresh ginseng is commonly processed through thermal treatment, which exerts a significant impact on its volatile compounds and a marked improvement in its flavor. Autoclaving is one of the important food processing technologies, which can effectively improve the flavor of fresh ginseng [6], facilitate food preservation, achieve sterilization, and retain the biological activity of ginseng [7]. This technology has been formally adopted in large-scale industrial production of fresh ginseng puree, instant ginseng functional food and ginseng fermentation substrates to inhibit mildew and microbial reproduction caused by high moisture of raw ginseng. Low-cost desktop autoclaves are accessible for small-scale processing laboratories and family-style ginseng processing workshops, indicating great application prospects for fresh ginseng preservation and deep processing. Existing studies have proven that autoclave treatment substantially reshapes ginseng phytochemical characteristics such as ginsenosides, terpenoids and phenolic antioxidants, and facilitates the transformation from protopanaxadiol and protopanaxatriol saponins to rare ginsenosides Rg3, Rh4 and Rg5 [8].

Currently, the majority of studies on ginseng have centered on the impacts of processing methods on bioactive constituents and their functional properties, while systematic analyses of processing-induced changes in ginseng volatile organic compounds (VOCs) and the consequential effects on flavor remain scarce. At present, E-nose, HS-SPME-GC-MS and HS-GC-IMS are important methods for odor analysis. Based on a bionic sensor array, E-nose can simulate biological olfaction to conduct overall response and pattern recognition for complex odors with a fast response speed [9]. HS-SPME-GC-MS combines the high-efficiency separation capability of gas chromatography with the accurate molecular structure identification capability of mass spectrometry, featuring simple operation, high sensitivity and fast analysis speed [10]. HS-GC-IMS integrates the separation dimension of chromatography with the rapid detection capability of ion mobility spectrometry at atmospheric pressure, boasting extremely high sensitivity and fast analysis speed, and is adept at the rapid and non-targeted screening of trace VOCs and dynamic process monitoring [11].

At present, most studies on ginseng focus on the effects of processing methods on bioactive components and their functional properties. Systematic analysis regarding the influence of processing on volatile substances in ginseng is still lacking, and the vital effect of processing on the flavor of fresh ginseng has been largely overlooked [12]. Herein, a combination of E-nose, HS-SPME-GC-MS and HS-GC-IMS was employed to comprehensively and systematically characterize the volatile compounds in autoclaved fresh ginseng. The findings are expected to provide a robust theoretical foundation for optimizing ginseng processing technologies, improving the flavor quality of ginseng products, and advancing the development of ginseng-based functional foods.

## 2. Materials and Methods

### 2.1. Materials

Fresh ginseng was purchased from local farmers in Jingyu County, Baishan, China.

### 2.2. Sample Pretreatment

Fresh three-year-old ginseng was purchased from local growers in Jingyu County, Baishan, China. Visible soil and residual root hairs on ginseng rhizomes were first rinsed rapidly with ultrapure water at room temperature, and discolored damaged parts were manually trimmed off. After surface moisture was absorbed with sterile filter paper, uniform ginseng roots were cut into thin slices with a sterile ceramic knife and fully homogenized. The homogenate was evenly divided into six equal independent aliquots, which constituted three biological replicate pairs. Three independent biological replicate samples were prepared for the fresh ginseng group (FG) and the autoclaved ginseng group (AG). Each biological sample underwent technical duplication during all instrumental detections, and all quantitative data presented in this manuscript are expressed as the average values of three biological replicates with standard deviation for statistical comparison. Three replicate samples were prepared and heated at 135 °C for 10 min in a high-temperature steam sterilizer to obtain three groups of fresh ginseng samples subjected to high-temperature and high-pressure treatment. Unheated ginseng tubers and the high-temperature- and high-pressure-treated ginseng samples were separately pulverized using a grinder to prepare test samples.

Volatile compounds were extracted and analyzed by GC-MS (Agilent Technologies, Santa Clara, CA, USA) following the established procedure. To evaluate instrumental repeatability and data stability, mixed quality control (QC) samples were prepared by pooling equal volumes of all sample extracts. One QC sample was injected every 6 sample vials during continuous detection. The peak area RSD of common characteristic volatiles in QC samples was controlled below 15%, which verified the reliability of the detection system. Each biological sample underwent technical duplication during all instrumental detections, and all quantitative data presented in this manuscript are expressed as the average values of three biological replicates with standard deviation for statistical comparison.

### 2.3. E-Nose Analysis

Electronic nose analysis was performed using a PEN3 electronic nose (Airsense, Schwerin, Germany). The electronic nose was equipped with a 10-sensor array for the analysis of diverse volatile components, whose performance is described in Table 1.

Samples were placed in beakers and sealed with sealing film. Headspace suction was applied by inserting the needle directly into the sampling vial. The measurement conditions were set as follows: sensor self-cleaning time, 80 s; sensor zeroing time, 5 s; sample preparation time, 5 s; sampling interval, 1 s; injection flow rate, 400 mL/min; and analytical sampling time, 80 s.

The E-nose adopted purified dry air and the instrument built-in sensor fingerprint database as dual reference benchmarks, and no concentration-dependent quantitative calibration curve was constructed in this study. For calibration operations, sensor baseline normalization was completed daily before batch sample testing; standard mixed gas was introduced monthly to correct long-term sensor signal drift. Three layers of verification were implemented to guarantee detection reliability: parallel blank matrix tests, fingerprint matching of standard mixed gas, and multivariate discrimination validation based on principal component analysis (PCA).

### 2.4. HS-SPME-GC–MS Analysis

HS-SPME-GC-MS analysis was conducted using an 8890-7000D GC-MS system (Agilent, Santa Clara, CA, USA). Samples (solid fresh samples by default unless otherwise specified) were retrieved from a −80 °C freezer, ground in liquid nitrogen, and homogenized by vortexing. Approximately 500 mg of each sample (1 mL for liquid samples) was weighed into a headspace vial, followed by the addition of saturated NaCl solution and 20 μL of internal standard solution (10 μg/mL). The mixture was then transferred to the SPME sampling vial. Subsequently, the vial was shaken for 5 min at a constant temperature of 60 °C. A 120 µm DVB/CWR/PDMS extraction fiber was inserted into the headspace of the sample vial for 15 min of headspace extraction, followed by desorption at 250 °C for 5 min prior to GC-MS separation and identification. Before sampling, the extraction fiber was conditioned at 250 °C for 5 min in a fiber conditioning station. Note: New extraction fibers were conditioned for 2 h in the fiber conditioning station before use. An SPME Arrow system was adopted, whose sensitivity is approximately 10 times that of conventional SPME fibers.

A DB-5MS capillary column (30 m × 0.25 mm × 0.25 μm, Agilent J&W Scientific, Folsom, CA, USA) was employed. The oven temperature program was set as follows: initial temperature 40 °C (held for 3.5 min), increased to 100 °C at 10 °C/min, further raised to 180 °C at 7 °C/min, and finally ramped to 280 °C at 25 °C/min and held for 5 min. High-purity helium (purity ≥ 99.999%) was used as the carrier gas at a constant flow rate of 1.2 mL/min. The injector temperature was 250 °C with splitless injection, and the solvent delay time was 3.5 min. An electron ionization (EI) source was applied with the ion source temperature at 230 °C, quadrupole temperature at 150 °C, and GC-MS interface temperature at 280 °C. The electron energy was 70 eV, and data were acquired in selected ion monitoring (SIM) mode for accurate qualitative and quantitative ion scanning (GB 23200.8-2016) [13].

A triple reference framework was integrated for the HS-SPME-GC-MS/MS platform, consisting of a self-built volatile substance mass spectrum library, constant concentration internal standard, blank ginseng matrix after nitrogen purging to remove inherent volatiles, and aqueous odor thresholds reported in published literatures for relative odor activity value (rOAV) calculation. Semi-quantitative calculation was realized via the peak area ratio of target compounds to internal standard; mixed quality control (QC) samples were inserted periodically during the whole batch test to monitor instrument response stability. Multi-dimensional validation methods were applied, including joint qualitative identification combining retention time and characteristic ions, data filtering based on coefficient of variation (CV) of QC samples, permutation test of orthogonal partial least squares discriminant analysis (OPLS-DA), and hierarchical classification of aroma contribution by rOAV value.

### 2.5. HS-GC-IMS Analysis

HS-GC-IMS analysis was performed using a Flavor Spec^®^ GC-IMS system (G.A.S. Gesellschaft für analytische Sensorsysteme mbH, Dortmund, Germany) coupled with an automatic headspace sampler. One gram of sample was weighed into a 20 mL headspace vial and incubated at 80 °C for 20 min before injection. Each sample was analyzed in triplicate. The experimental conditions were as follows: injection volume 500 μL; incubation time 20 min; splitless injection; syringe temperature 85 °C; incubation agitation speed 500 r/min; and column temperature 60 °C.

For volatile component determination, high-purity nitrogen (purity ≥ 99.999%) was used as the carrier gas. The initial column flow rate was 2.0 mL/min (held for 2 min), linearly increased to 10.0 mL/min within 8 min, further ramped to 100.0 mL/min within 10 min, and held for 40 min. The total chromatographic run time was 60 min, and the injector temperature was 80 °C. A tritium (^3^H) ionization source was used with a drift tube length of 53 mm, electric field intensity of 500 V/cm, and drift tube temperature of 45 °C. High-purity nitrogen (purity ≥ 99.999%) was applied as the drift gas at a flow rate of 75.0 mL/min in positive ion mode.

A multi-dimensional qualitative system was constructed with C_4_~C_9_ n-ketone mixed standard solution, built-in NIST mass spectrum library and reaction ion peak (RIP) as unified chromatogram alignment markers. For calibration, the linear retention index curve was fitted based on ketone mixed standards; routine calibration of the drift tube was performed daily. Relative quantification between groups was achieved via peak area extraction of two-dimensional spectra, and gradient standard solutions were not adopted for absolute quantification in this research. The reliability of GC-IMS results was verified by dual-parameter matching of retention index and normalized drift time, three technical replicates of each sample, and comparative analysis of differential spectra between groups.

### 2.6. Data Procession

VOCal 2.3 data processing software was applied to generate 3D spectra, 2D spectra, difference spectra, fingerprint spectra and PCA plots for the comparison of VOCs among samples [14]. Unit variance scaling (UV) was used for the processing of PCA and heatmap data, and MetaboAnalystR 4.0 was adopted for orthogonal partial least squares discriminant analysis (OPLS-DA) [15]. All data sets were statistically analyzed where means were expressed in mean ± SEM. Analysis of variance (ANOVA) comparisons were performed to compare the means of the model group and the treatment groups.

ANOVA calculation was implemented on raw abundance data of three biological replicates (*n* = 3) from the FG and AG groups. Fold change, *p*-value and FDR values for differential volatile metabolites were all calculated based on averaged VOC relative abundance instead of single-sample measurement data. All tabulated quantitative results follow the standard statistical note: Data are mean ± standard deviation (*n* = 3); data in the same column with different superscripts are significantly different (*p* < 0.05).

## 3. Results and Analysis

### 3.1. E-Nose-Based Sensory Difference Analysis

**Figure 1:**
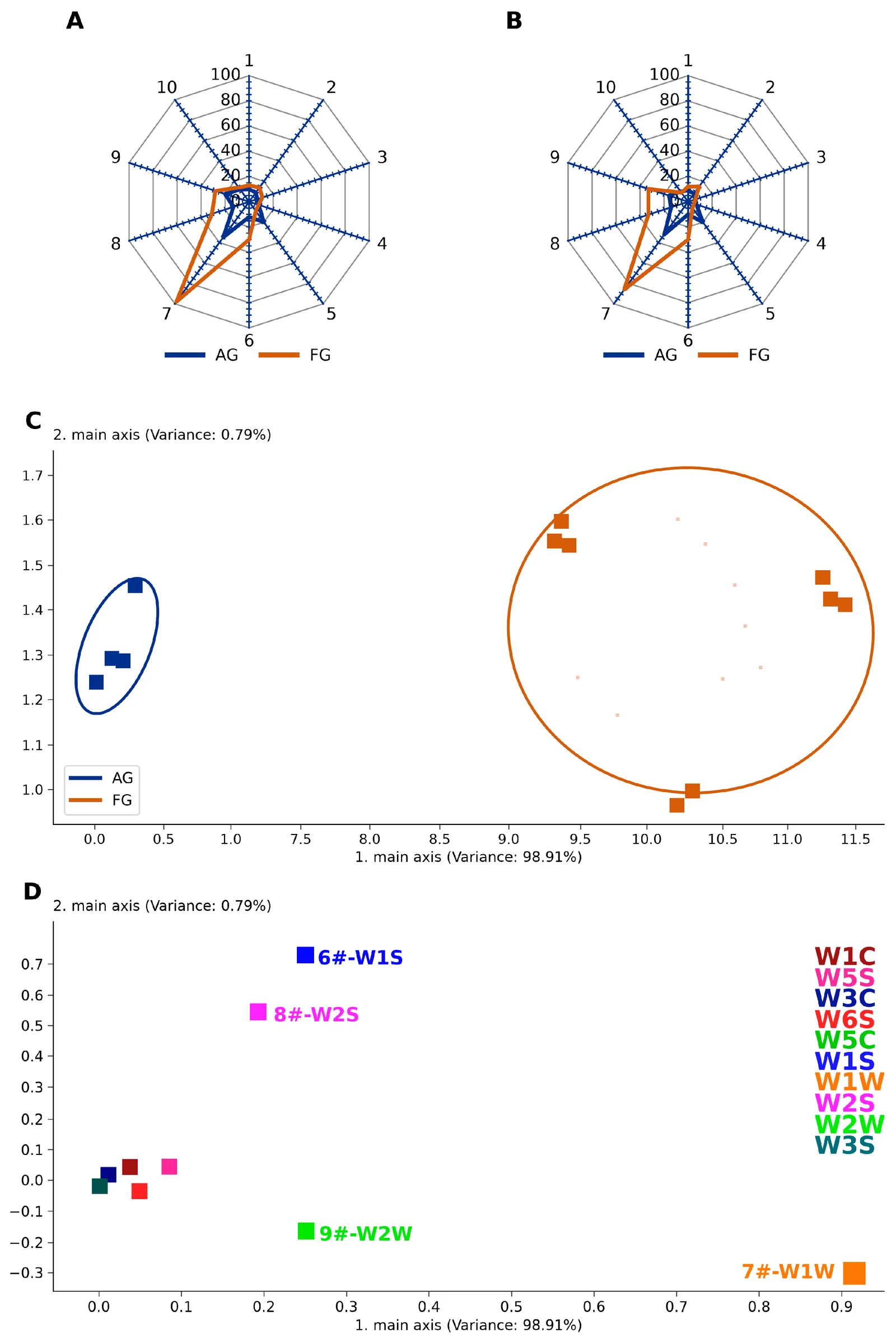
Analysis chart of peak value comparison data of ginseng samples (**A**). Comparative data analysis chart of 71 s to 73 s average values (**B**). PCA principal component analysis chart (**C**). Loading sensor contribution analysis chart (**D**). Note: Data are mean ± standard deviation, *n* = 3 biological replicates for FG and AG separately; distinct superscript letters within identical axes denote significant differences at *p* < 0.05.

E-nose uses metal oxide sensors to classify samples efficiently and rapidly according to characteristic flavor compounds and to characterize the overall VOC information of samples quickly. Each sensor is sensitive to a certain type of compound, and the resistance ratio of the sensor represents the response value of VOCs [16]. FG and AG groups each contained three independent biological replicates (*n* = 3). All sensor response values plotted in radar graphs, PCA and loading charts are the average values of three parallel samples with standard deviation for inter-group significance evaluation. Odor information of the samples was collected by E-nose and analyzed by the self-contained analysis software of E-nose. PCA, linear discriminant analysis (LDA) and sensor loading analysis were used to analyze the odor differences between samples, and the results are shown in Figure 1. For FG and AG group, the E-nose sensors responded normally to their odors, with the maximum response value obviously within 150. The odor intensity decreased significantly after autoclaving. As shown in Figure 1A,B, there were significant differences between FG and AG. Comparison of the response values at the peak and stable stages revealed that sensors No. 7, No. 6, No. 8 and No. 9 exhibited large differences in response values, indicating that the contents of short-chain alkanes, inorganic sulfides, alcohols, ethers, aldehydes, ketones and organic sulfides in ginseng increased significantly after autoclaving.

To further determine the effects of autoclaving on ginseng, PCA was performed on the data. As depicted in Figure 1C, the variance contribution rates of the first principal component (PC1) and the second principal component (PC2) were 85.23% and 8.4674%, respectively, with a total contribution rate of nearly 93.698%, which indicated that the principal components reflected the overall characteristics of most samples, and the differences between different samples were mainly reflected in PC1. As illustrated in Figure 1C, the AG and FG sample groups exhibit no overlap and are distinctly separated, indicating significant inter-group differences and considerable variations in their flavor profiles.

Loading analysis reveals the main volatile components responsible for the changes in sample components by determining the contribution rate of each sensor to the changes in sample components [17]. The results of loading analysis in Figure 1D show that the sensors mainly responding to FG and AG were No. 7, No. 6, No. 9, No. 2 and No. 8. Sensor No. 7 had the highest contribution rate to PC1, and sensor No. 6 had the maximum contribution rate to PC2. These results indicate that short-chain alkanes such as methane and organic sulfides could be used as important indicators of flavor differences. Compared with FG, AG had a lower content of inorganic sulfides and contained more abundant short-chain alkanes such as methane, organic sulfides and aromatic components.

### 3.2. Characterization of Volatile Compounds via HS-SPME-GC–MS

#### 3.2.1. Identification and Quantification of Metabolites

**Figure 2:**
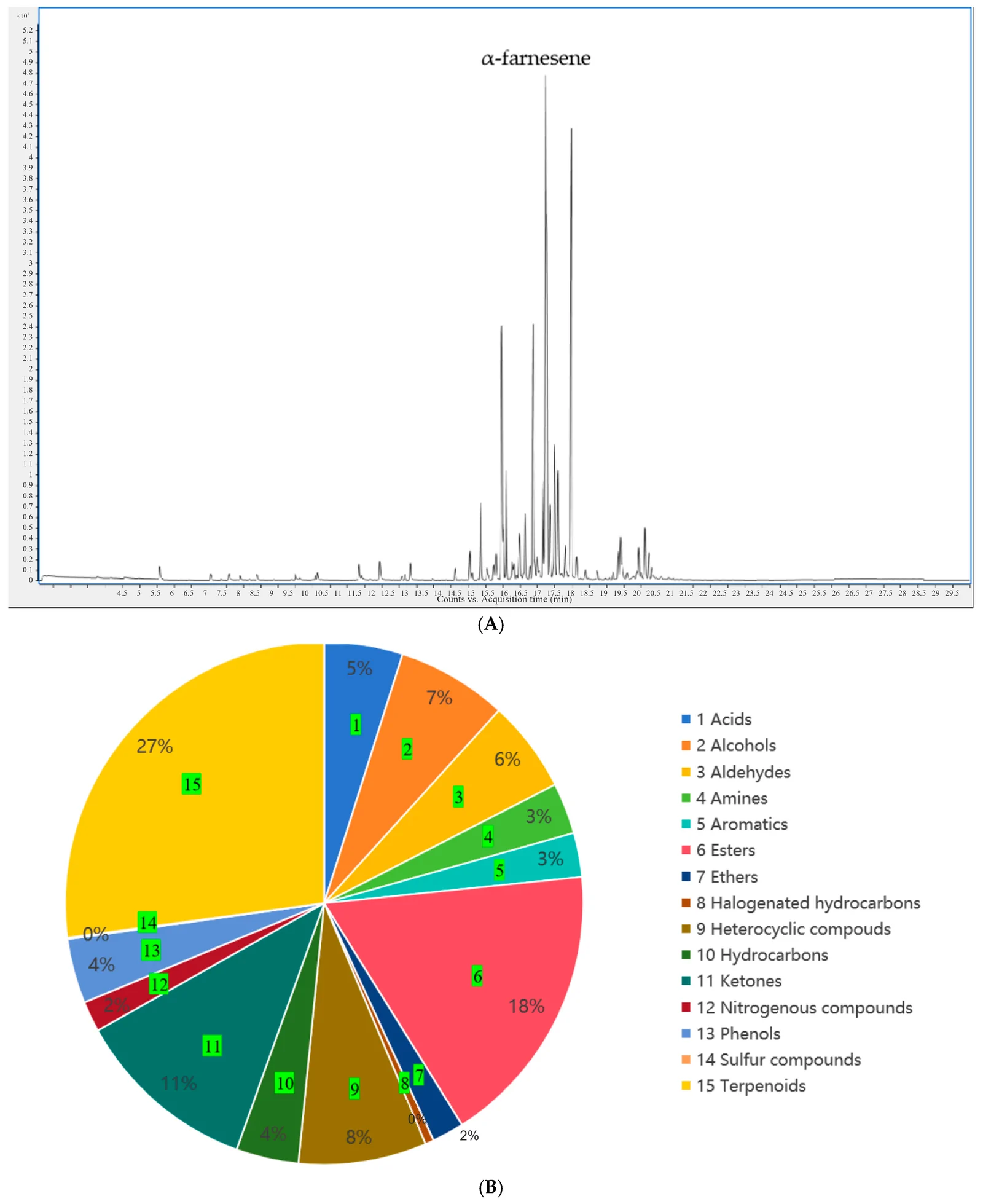

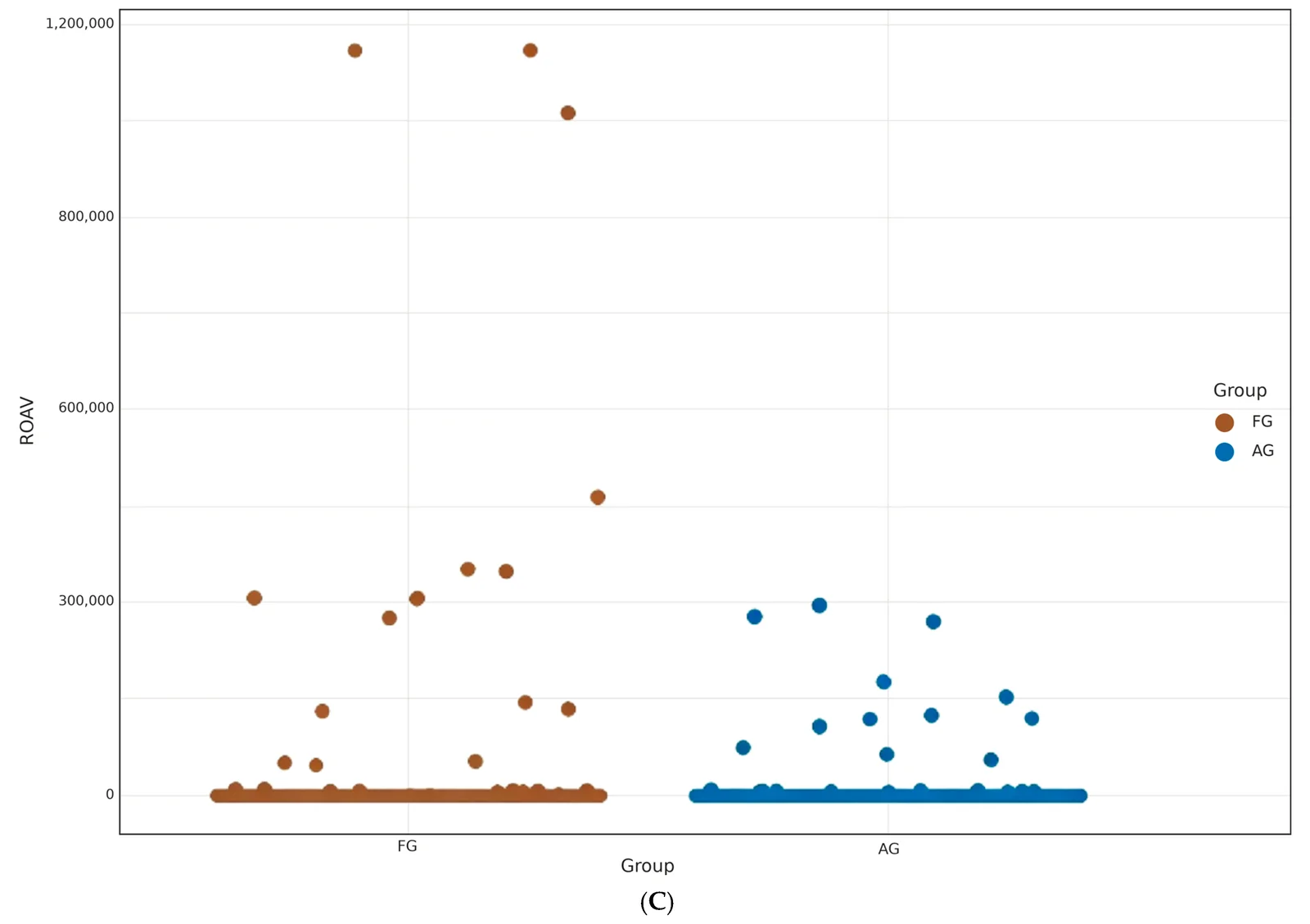
Total ion chromatogram of mixed sample quality analysis (**A**). Metabolite category composition pie chart (**B**). ROAV odor activity value scatter plot (**C**). The dominant chromatographic peak shown in the TIC profile (**A**) corresponds to α-farnesene, which is the most abundant terpenoid volatile compound in ginseng.

A total of 1103 metabolites were detected by widely targeted metabolomics-based GC-MS, among which 253 metabolites were significantly different, including 181 metabolites with decreased contents and 72 with increased contents (Figure 2). As shown in Figure 2A, the total ion current chromatograms overlapped substantially, with consistent peak intensities at identical retention times. This result demonstrates excellent signal stability of the mass spectrometer during repeated analyses of the same sample, as well as reliable data and good experimental reproducibility.

Metabolite composition presented obvious sample specificity, with distinct differences in metabolite categories and relative contents among various sample types [18]. As illustrated in Figure 2B, profiling the composition and relative abundance of metabolites facilitates a comprehensive assessment of the overall distribution of major metabolites across all samples. A total of ten metabolite classes were detected in FG and AG samples, including terpenoids, hydrocarbons, alcohols and aldehydes. Terpenoids dominated in relative content and were responsible for the woody aroma of ginseng [19], while esters, the second most abundant group, contributed to the fruity and sweet notes of ginseng [12].

The relative odor activity value (rOAV) is a typical and effective method for identifying key flavor compounds in food established by combining the sensory threshold of compounds [20], which is used to clarify the contribution of each aroma compound to the overall aroma characteristics of the samples. The rOVE is expressed as the ratio of the concentration of volatile flavor substances to their own odor threshold (aqueous phase). If the rOAV of a compound exceeds its odor threshold, the compound contributes to the sample aroma. Generally, a higher rOAV indicates a stronger effect on the overall flavor. Relative odor activity value (rOAV) screening identified a total of 77 volatile compounds that dominantly contribute to the integrated aroma profile of ginseng. Terpenes and terpenols represented by α-farnesene, E-β-farnesene, d-menthol, carotol, limonene and α-pinene, conjugated unsaturated enals and linear saturated aldehydes exemplified by E-2-hexenal, (E,E)-2,4-octadienal, hexanal and heptanal, as well as intrinsic aliphatic alcohols including 1-hexanol and 1-octen-3-ol, alongside natural short-chain fruit esters such as isoamyl acetate and cis-3-hexenyl isovalerate, were markedly depleted upon high-temperature high-pressure hydrothermal treatment. The thermally induced attenuation of these native volatile fractions arises from multiple synergistic pathways: unsaturated monoterpenes and sesquiterpenes undergo oxidative cleavage and ring-opening rearrangement under coupled thermal and aqueous stress; carbonyl groups of endogenous aldehydes readily engage in preliminary Maillard condensation reactions with free amino residues within ginseng matrix, resulting in continuous substrate consumption; native alcohols are dual-depleted via esterification with thermally liberated fatty acids and steam-mediated volatilization loss, while intrinsic esters suffer hydrolytic dissociation of ester linkages under high-moisture sterilization conditions. Collectively, these native volatiles constitute the characteristic grassy, earthy and sharp resinous woody base aroma of raw fresh ginseng, and their pronounced degradation substantially mitigates the pungent green sensory attributes of autoclaved ginseng [6,12,21]. In contrast, thermally derived aromatic aldehydes (furfural, phenylacetaldehyde, citronellal, nonanal), saturated hydroxyketones and alkyl ketones (3-hydroxy-2-butanone, 2-decanone, 2-nonanone), nitrogen-containing heterocycles (2,3-diethyl-5-methylpyrazine, pyridine), sulfur-bearing volatile metabolites (dimethyl disulfide, dimethyl trisulfide), furanones and aromatic esters (5-heptyldihydro-2(3H)-furanone, methyl 2-(acetylamino)benzoate), alongside isomerized sesquiterpenoids (δ-cadinene, β-elemene), were significantly accumulated in AG samples. Autoclaving facilitates the hydrolytic depolymerization of ginseng polysaccharides to release abundant reducing sugars, simultaneously disrupting cellular compartments to accelerate the solubilization of endogenous free amino acids; elevated temperature and humidity synergistically amplify the reaction kinetics of Maillard cascade and Strecker degradation, while thermal cracking of fatty acids provides carboxylic substrates for thermally driven esterification. Meanwhile, labile native terpenoids undergo intramolecular cyclization and isomerization to generate thermodynamically stable sesquiterpene derivatives. The coordinated progression of the aforementioned thermochemical reactions enriches a spectrum of volatile compounds conferring roasted, creamy, caramel-like and nutty olfactory notes, which endow AG with mild, mellow woody undertones and comprehensively reshape the holistic volatile fingerprint of ginseng. The bidirectional transformation pattern of ginseng volatile flavor metabolites triggered by autoclaving observed herein aligns well with previous investigations regarding aroma variations of ginseng subjected to diverse thermal dehydration and autoclave sterilization of aromatic spices [8,12,21].

#### 3.2.2. Quality Control Analysis of Samples

**Figure 3:**
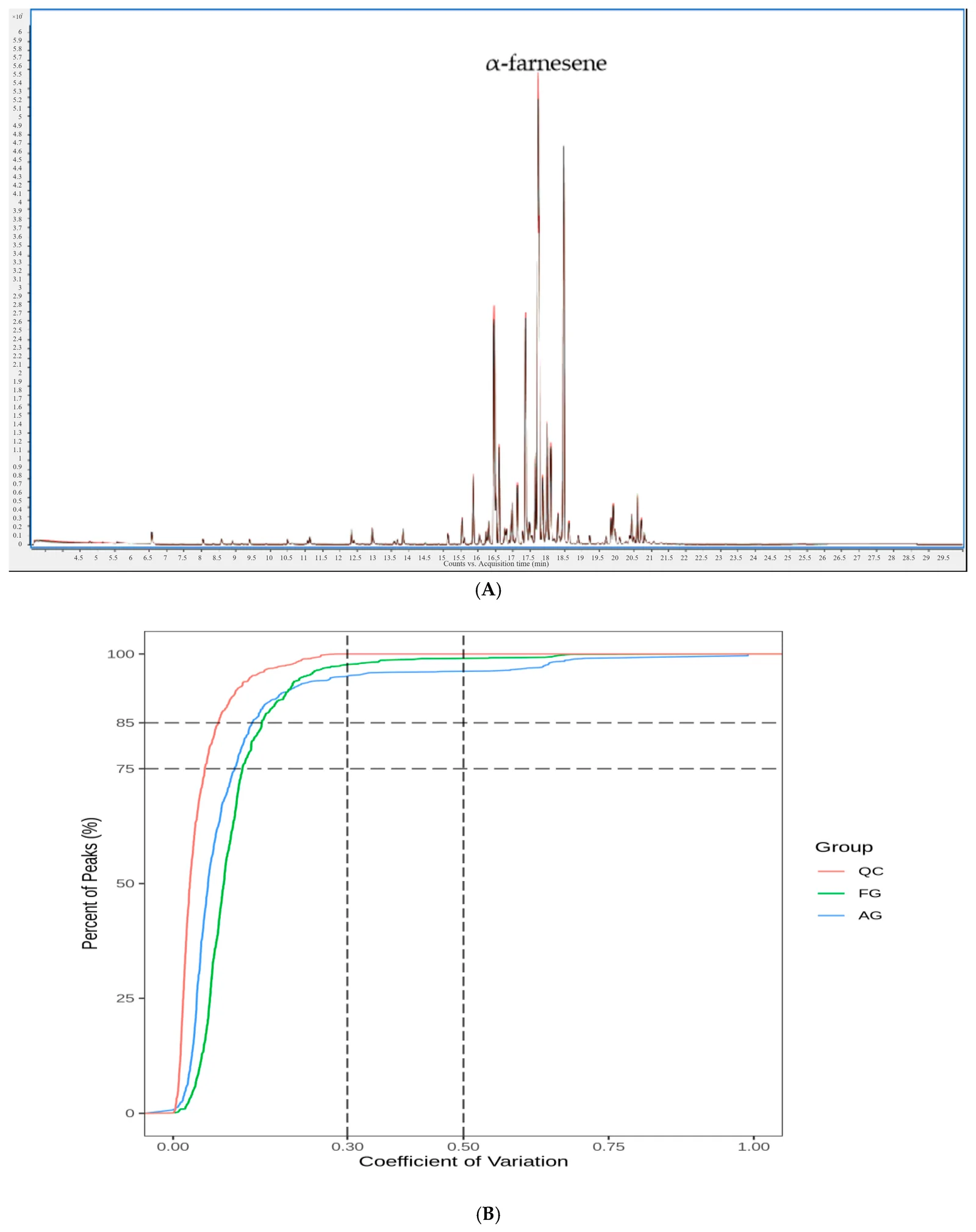

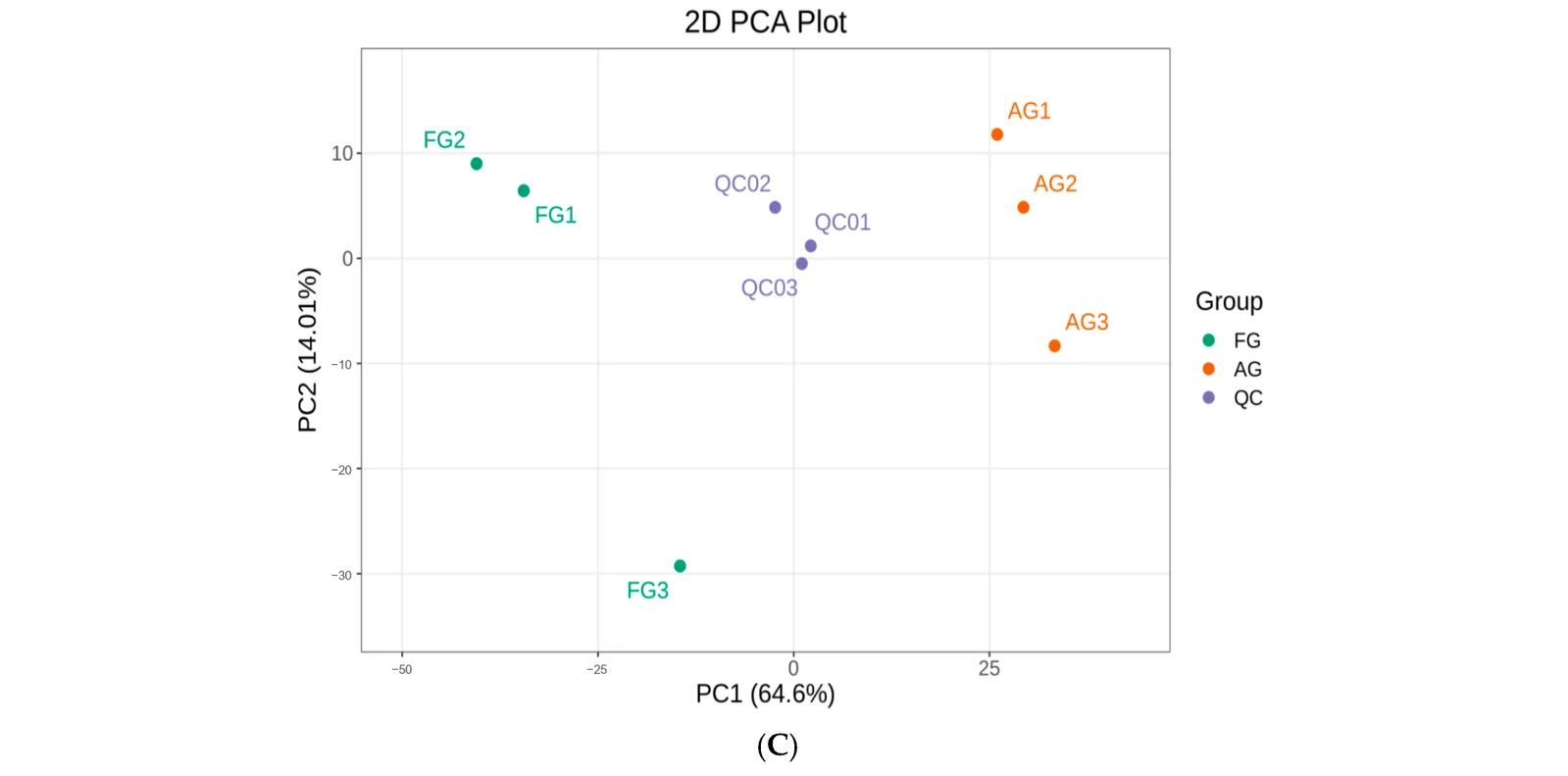
QC sample mass spectrometry TIC overlay (**A**). CV distribution chart of each group of samples (**B**). PCA score plot of the mass spectrometry data of each sample group and the quality control samples (**C**). The dominant highest chromatographic peak in TIC overlay (**A**) is annotated as α-farnesene, which is the most abundant terpene volatile in ginseng. Note: *n* = 3 biological replicates for FG and AG groups; all volatile abundance data plotted are mean ± standard deviation, significant differences were judged by *p* < 0.05 with different superscript labels.

Quality control (QC) samples were prepared by mixing all samples and used to analyze the repeatability of samples under the same processing method [22], and the results are shown in Figure 3. The total ion current chromatograms of QC samples showed a high degree of overlap, indicating high instrument stability, which provided an important guarantee for the repeatability and reliability of the data. In Figure 3B, the coefficient of variation (CV) is an important index to evaluate data stability and repeatability. The proportions of CV values less than 0.3 in QC samples and experimental groups were both higher than 75%, indicating that the experimental data were extremely stable. The distribution of CV curves of AG and FG groups reflected the fluctuation of flavor substances in the samples. At the same CV value, the proportion of volatile substances in AG group was higher than that in the FG group, which further indicated that autoclaving had an improvement effect on the volatilization of flavor substances.

PCA score plots can intuitively reflect the degree of separation between and within sample groups [23]. As shown in Figure 3C, the AG and FG groups showed an obvious separation trend in the PCA score plot, with large inter-group differences and small intra-group differences, indicating significant differences in volatile metabolites between FG and AG. The contribution rates of PC1 and PC2 were 64.6% and 14.01%, respectively, indicating that these components had an extremely high contribution rate to inter-group differences, which further verified the significant differences in the composition of volatile metabolites between FG and AG.

#### 3.2.3. Differential Component Analysis of Different Samples

**Figure 4:**
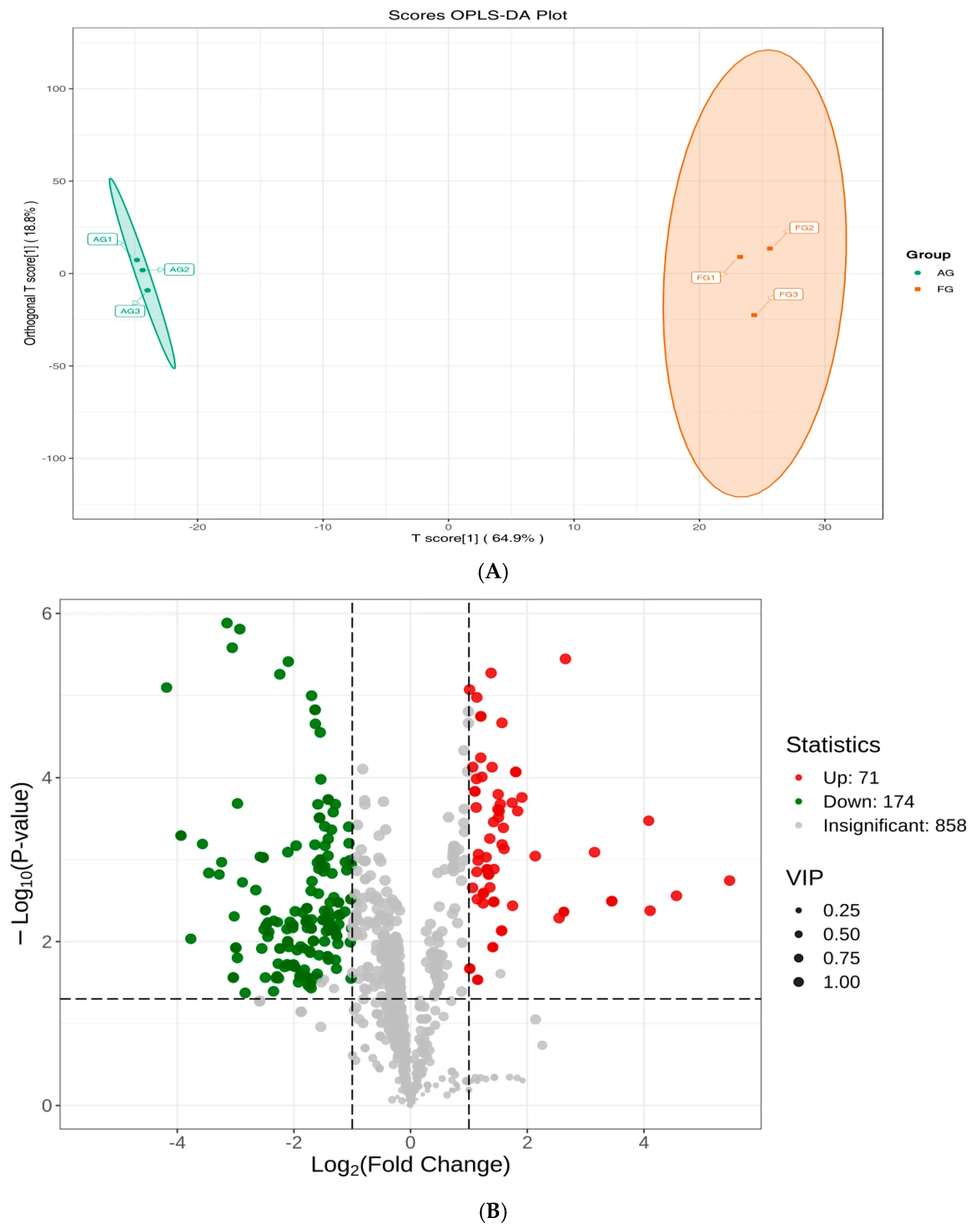

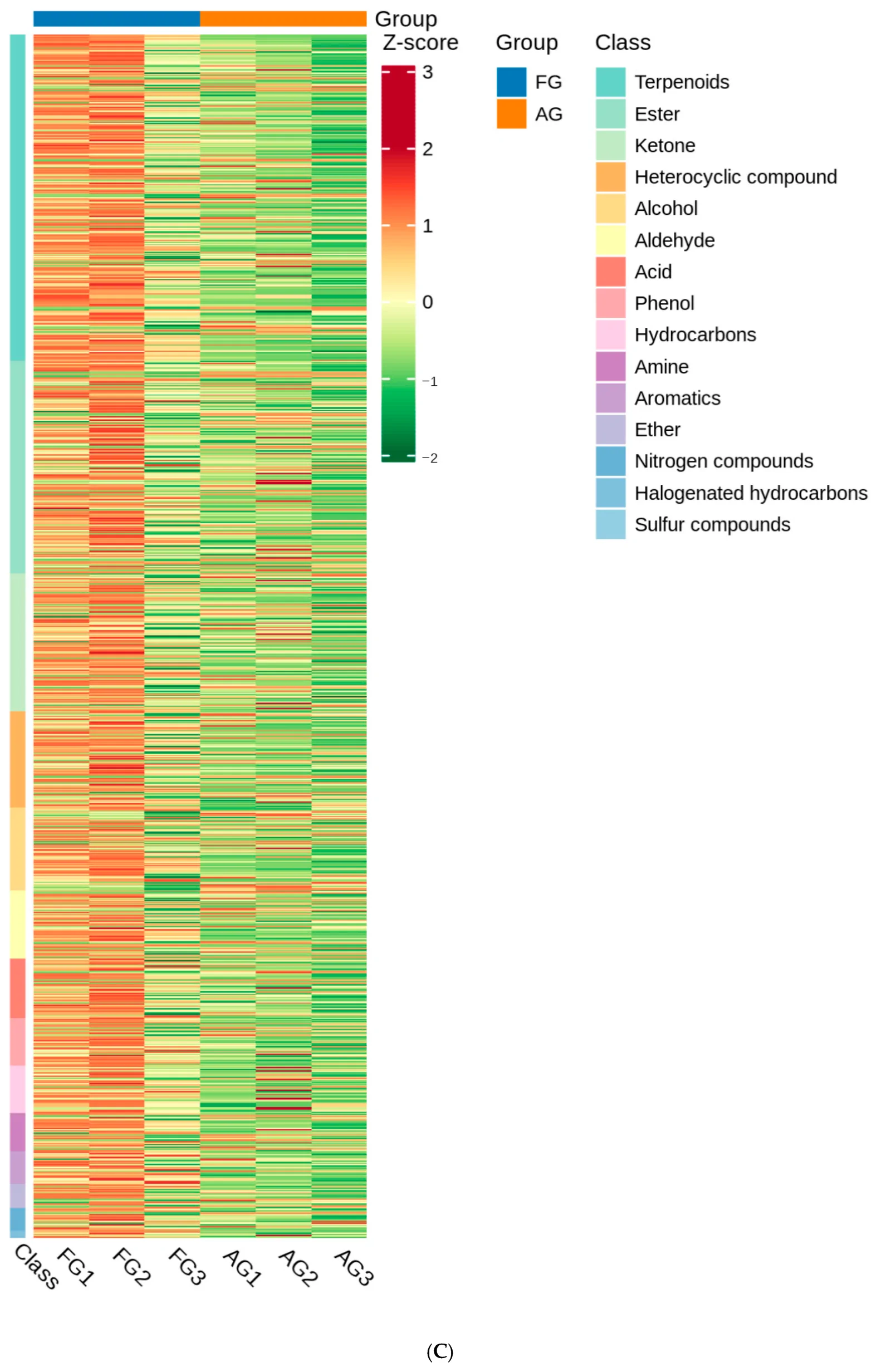
QC sample mass spectrometry TIC overlay (**A**). CV distribution chart of each group of samples (**B**). PCA score plot of the mass spectrometry data of each sample group and the quality control samples (**C**). Note: Each group has 3 biological replicates (*n* = 3); all metabolite abundance data are mean ± standard deviation.

An OPLS-DA model was used to analyze the metabolomic data, and score plots of each group were drawn to further show the differences between groups (Figure 4). The model interpretation parameters were R2X = 0.837 and R2Y = 0.999, and the model prediction ability parameter was Q2 = 0.925. FG and AG samples were clustered in their respective regions with obvious classification. To further screen the core differential volatile metabolites between FG and AG, the top 20 key VOCs ranked by VIP value are summarized in Table 2.

Hierarchical clustering was performed on the differential metabolites in the FG and AG samples, and the similarities and differences between different samples were presented by a cluster heatmap. In Figure 4C, the ordinate represents the relative contents of 15 flavor compounds with rOAVs higher than the VOCs threshold, in which red and blue represent the contents of different substances. The deeper the blue color, the lower the content of the compound, and the deeper the red color, the higher the content. The key volatile substances in the AG and FG samples showed good clustering, and there were obvious clustering differences in the composition and content of flavor metabolites between the AG and FG samples. Metabolites such as terpenoids, esters and ketones were identified as the key metabolite classes, with remarkable differences between the two groups, indicating that autoclaving had an obvious impact on the content and composition of volatile metabolites in ginseng, which provided a basis for the subsequent analysis of differential metabolites.

### 3.3. Analysis of HS-GC-IMS Results

**Figure 5:**
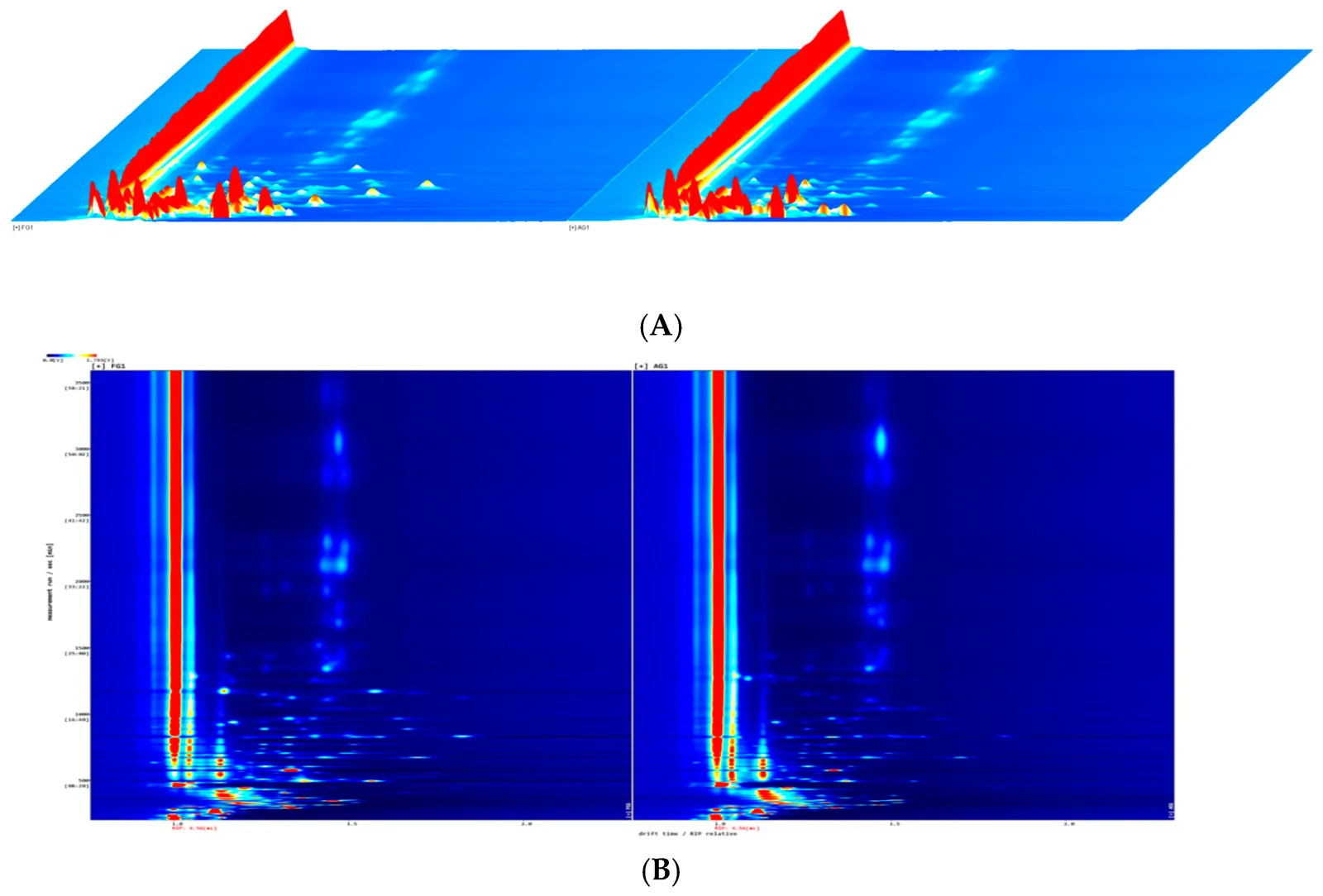

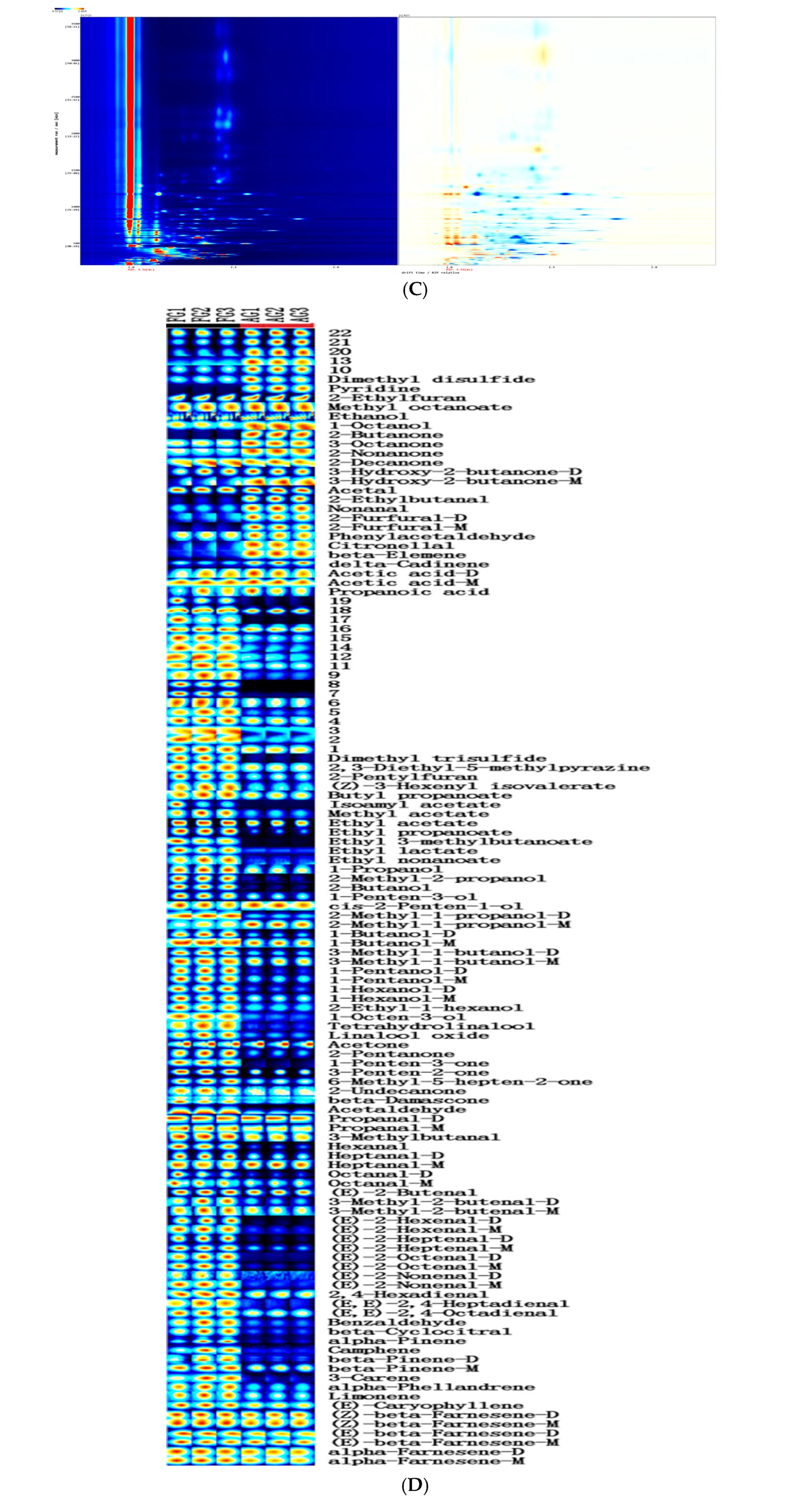
Three-dimensional GC-IMS spectra of volatile components in FG and AG samples (**A**). GC-IMS 2D spectra of volatile components in FG and AG samples (**B**). GC-IMS difference spectra of volatile components in FG and AG samples (**C**). GC-IMS fingerprint profiles of volatile components in FG and AG samples (**D**).

Six groups of samples were detected by GC-IMS. According to the retention time and migration time of volatile substances in each sample, the 3D GC-IMS spectra and top views of the six groups of samples were obtained by comparison with the built-in volatile substance database of GC-IMS (Figure 5). Each peak and each point on both sides of the RIP peak in the figure represents a VOC, and the color value indicates the concentration of VOCs, with the color reflecting the peak intensity of the substance. From blue to red, the deeper the color, the higher the peak intensity.

Figure 5A,B intuitively show that there were certain differences in VOCs between different samples. To further compare the differences in volatile components between samples intuitively, the spectrum of FG was selected as the reference, and the spectrum of AG was subtracted from the reference to obtain the difference comparison map of different samples (Figure 5C). The background after subtraction was white, red indicated that the concentration of the substance in the target sample was higher than that in the reference, and blue indicated that the concentration of the substance in the target sample was lower than that in the reference. These results indicated that the flavor substances of ginseng underwent significant dynamic changes during autoclaving, and the signal intensity of AG was higher than that of FG.

Fingerprint analysis was performed on all volatile substances to further compare the volatile substances in the samples (Figure 5D). The results showed that FG had higher contents of α-farnesene, E-β-farnesene, Z-β-farnesene, E-caryophyllene, limonene, α-phellandrene, 3-carene, β-pinene, camphene, α-pinene, β-cyclocitral, benzaldehyde, (E,E)-2,4-octadienal, (E,E)-2,4-heptadienal, 2,4-hexadienal, E-2-nonenal, E-2-octenal, E-2-heptenal, E-2-hexenal, 3-methyl-2-butenal, E-2-butenal, octanal, heptanal, hexanal, 3-methylbutanal, propanal, acetaldehyde, β-damascenone, 2-undecanone, 6-methyl-5-hepten-2-one, 3-penten-2-one, 1-penten-3-one, 2-pentanone, acetone, linalool oxide, tetrahydrolinalool, 1-octen-3-ol, 2-ethyl-1-hexanol, 1-hexanol, 1-pentanol, 3-methyl-1-butanol, 1-butanol, 2-methyl-1-propanol, Z-2-penten-1-ol, 1-penten-3-ol, 2-butanol, 2-methyl-2-propanol, 1-propanol, ethyl nonanoate, ethyl lactate, ethyl 3-methylbutanoate, ethyl propionate, ethyl acetate, methyl acetate, isoamyl acetate, butyl propionate, cis-3-hexenyl isovalerate, 2-pentylfuran, 2,3-diethyl-5-methylpyrazine, dimethyl trisulfide and other substances. AG had higher contents of propanoic acid, acetic acid, δ-cadinene, β-elemene, citronellal, phenylacetaldehyde, 2-furfural, nonanal, 2-ethylbutanal, acetal, 3-hydroxy-2-butanone, 2-decanone, 2-nonanone, 3-octanone, 2-butanone, 1-octanol, ethanol, methyl octanoate, 2-ethylfuran, pyridine, dimethyl disulfide and other substances.

### 3.4. Characterization and Qualitative Analysis of Volatile Profiles

**Figure 6:**
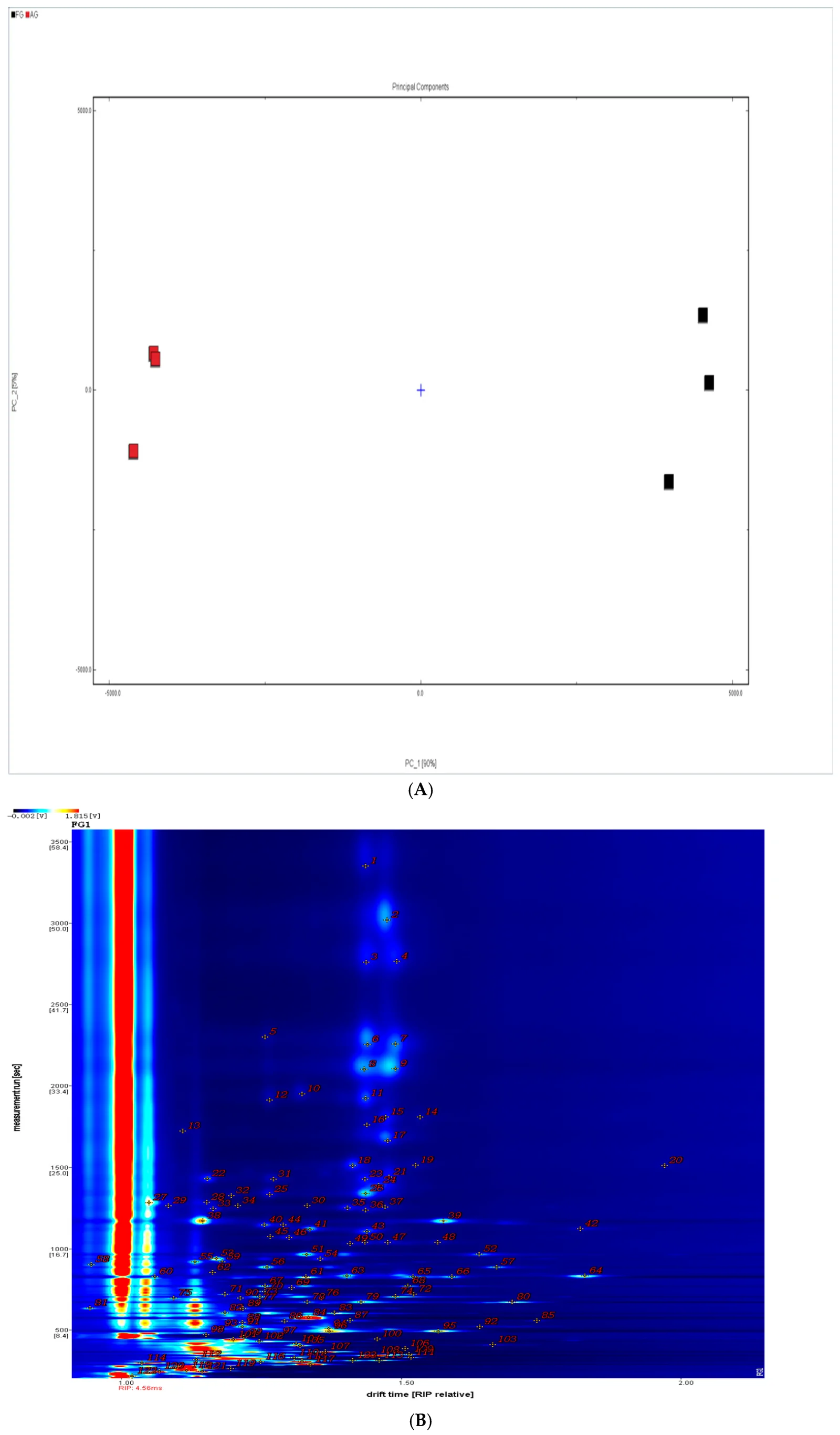

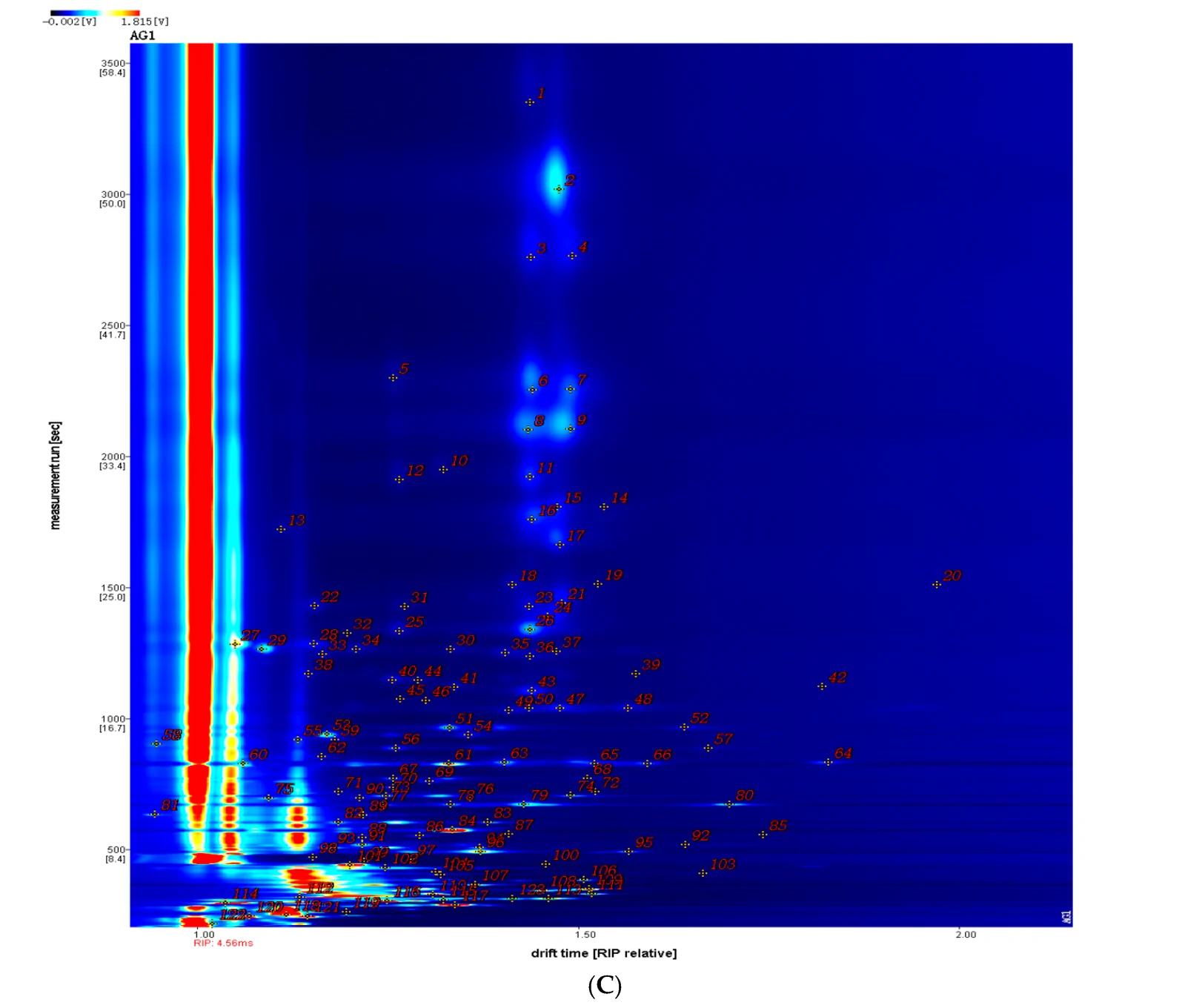
PCA score plot of volatile components in FG and AG samples (**A**). Qualitative spectrum of GC−IMS (**B**,**C**). Note: FG *n* = 3, AG *n* = 3; all GC−IMS signal intensities are mean ± standard deviation of three biological replicates, significant differences (*p* < 0.05) are distinguished by different superscript symbols.

As shown in Figure 6A, the contribution rates of PC1 and PC2 were 90% and 5%, respectively, with a total contribution rate of 95%, indicating that PC1 and PC2 reflected most of the sample information. Moreover, different ginseng samples were clearly separated and the samples in the same group were clustered together, indicating significant inter-group differences and good intra-group repeatability.

A total of 81 volatile components were qualitatively identified in the FG and AG samples by GC-IMS (Figure 6B,C). Further comparison of the spectral differences between FG and AG revealed that some spots only appeared in the FG or AG group, indicating that autoclaving led to the production or disappearance of volatile substances in ginseng. The color intensity in Figure 6 reflects the response intensity of volatile components, which indicates the effects of autoclaving on volatile substances in ginseng. Detailed qualitative information, including CAS number, molecular formula, retention time and drift time of all 101 volatile compounds identified by HS-GC-IMS, is displayed in Table 3.

## 4. Discussion

As a traditional plant with both medicinal and edible values, ginseng exerts multiple biological effects such as anti-inflammatory, antioxidant, anti-tumor, immunomodulatory and neuroprotective effects, and is gradually being applied in the development of functional foods. Importantly, the flavor of ginseng directly affects consumer acceptance and product quality. Fresh ginseng has a high water content and strong endogenous hydrolase activity, making it highly susceptible to microbial contamination, degradation and mildew, which seriously affects its quality and efficacy [5]. Therefore, accordingly, appropriate processing treatments can guarantee the quality of ginseng products, among which hot air drying, freeze drying, sun drying and high-pressure steam sterilization are widely adopted. For ginseng treated by hot air drying, the contents of some aldehydes and ketones decrease due to prolonged oxidation [21]. Vacuum freeze drying can well retain the original color, aroma, taste and heat-sensitive components of ginseng; nevertheless, this technique requires expensive equipment and consumes excessive energy, making it difficult to realize large-scale production [24]. Compared with conventional atmospheric steaming, autoclave treatment under 135 °C high temperature and high pressure significantly altered the overall volatile profile of ginseng [12]. The high-pressure environment accelerates thermal degradation, oxidation and cyclization reactions of terpenoids, esters and ketones, leading to an obvious accumulation of heat-generated volatile products in the AG samples [8].

Currently, hot air drying, freeze drying, and sun drying are widely employed processing methods for ginseng. Nevertheless, the contents of some aldehydes and ketones in ginseng processed by hot air drying decrease due to long-term oxidation [21]. Vacuum freeze drying can well preserve the original color, aroma, taste and heat-sensitive components of ginseng, but it requires expensive equipment and high energy consumption, making it difficult for large-scale production [22]. In contrast, as an efficient and reliable food processing technology, autoclaving can not only achieve complete sterilization and enzyme inactivation and retain the biological activity of ginseng to the greatest extent [7], but also significantly improves the flavor of fresh ginseng. Ding et al. [25]. systematically investigated the flavor modification of American ginseng via single lactic acid bacteria, single yeast and mixed fermentation, and revealed fundamental differences between microbial fermentation and high-pressure autoclaving in reshaping ginseng aroma. Mixed fermentation mainly produced abundant fruit-like ester substances and organic acids to mask the inherent bitter taste of ginseng, while barely generating sulfur-containing volatile compounds. Its characteristic key aroma substances included octanal, linalool and γ-decalactone with citrus and apricot notes. In contrast, autoclaving depends on high-pressure thermal chemical reactions (Maillard reaction, terpene thermal cleavage) rather than microbial metabolism, producing massive organic sulfides, furans and roasted aroma compounds such as 2-furfural, which endow ginseng with mellow woody and caramelized sensory characteristics. The two processing routes form distinct flavor regulation systems: fermentation is suitable for developing light fruity ginseng products, while autoclaving targets mellow roasted flavor instant ginseng puree and functional food substrates. Lee et al. [26]. adopted metal oxide-based E-nose and GC-MS to track aroma changes during red ginseng production, and verified that repeated steaming/drying eliminated fresh ginseng’s herbal and floral volatiles, while generating maltol (3-hydroxy-2-methyl-pyran-4-one) as the signature sweet roast volatile of processed red ginseng. Consistent with this conclusion, our HS-GC-IMS results also detected significantly elevated furans, aldehydes and short-chain organic acids in AG, such as 2-furfural, phenylacetaldehyde and 3-hydroxy-2-butanone—typical Maillard and Strecker degradation products triggered by high heat and pressure. Unlike conventional atmospheric steaming (100 °C, 2 h) in red ginseng preparation, the 135 °C autoclaving condition in the present study created a stronger thermal field that accelerated terpene degradation and esterification simultaneously: HS-SPME-GC-MS quantified 181 downregulated volatile metabolites (dominated by raw sesquiterpenes, including α-farnesene, caryophyllene) and 72 upregulated flavor compounds (various aliphatic esters and sulfur-containing organics). This observation aligns with Zhang et al. [8], who reported that commercial high-pressure sterilization facilitated the conversion of protopanaxadiol and protopanaxatriol ginsenosides into rare saponins, and our study further supplements the volatile flavor dimension of this thermal transformation mechanism.

Currently, most studies on volatile flavor substances of ginseng adopt a single detection technology to analyze the volatile components of ginseng. Wang et al. [27]. analyzed the flavor differential substances of 12 different kinds of ginseng by HS-SPME-GC-MS and found that ginseng had the highest contents of terpenoids, alcohols and aldehydes, but the effects of processing methods on flavor was not detected and failed to fully cover flavor substances with different contents. Cui et al. [28]. combined E-nose with GC-MS to identify ginseng of different ages and identified 91 volatile components, but the variance explanation rate of E-nose PCA was 85.51%, which was lower than that in this study, and the trace components were not further analyzed. Zhang et al. [29]. analyzed the flavor substances of sun-dried ginseng and fresh ginseng based on sensory evaluation and E-nose technology, and confirmed that processed ginseng had a richer flavor, but only E-nose was used for the sensory clustering of volatile components, resulting in a single research dimension. Xiang et al. [21] used GC-IMS for the rapid analysis of volatile substances in ginseng processed by different drying methods, identified 72 volatile substances and found that volatile substances in ginseng varied with different drying methods, but the changes of ginseng flavor substances under drying conditions can not be comprehensively analyzed. Cho et al. [30]. utilized jet impingement fluidized bed roasting technology to roast red ginseng lateral roots under segmented dry heating at 150–200 °C for 10 min and investigated the influence of roasting temperature on aroma profiles. They verified that high-temperature roasting massively produced maltol and furfural via the Maillard browning reaction, which is consistent with the formation rule of thermally induced aroma compounds observed in autoclaved ginseng in the present study. Their results showed that maltol content increased by more than 10 times after roasting at 170 °C, and furfural content rose over 444-fold at 200 °C compared with untreated red ginseng. Nevertheless, the jet impingement fluidized bed only relies on dry hot air without a high-temperature saturated steam environment, so it cannot trigger extensive cleavage of sesquiterpenes and accumulation of sulfur-containing volatile compounds as observed in the autoclaved ginseng of this study. Meanwhile, dry roasting leads to remarkable losses of medium- and long-chain ester volatiles, whereas the moist high-pressure conditions adopted in our study facilitate esterification reactions, rendering aliphatic esters the most significantly upregulated differential metabolites. Furthermore, Cho’s research only adopted gas chromatography–mass spectrometry to quantify several typical roasted aroma compounds, without multi-dimensional fingerprint characterization combining electronic nose and GC-IMS, lacking data support for integral odor discrimination and rapid screening of trace volatile substances. In the present study, E-nose, HS-SPME-GC-MS and HS-GC-IMS were combined to analysis the effect of autoclaving on the flavor of fresh ginseng. Based on bionics principles, E-nose can conduct rapid response and pattern recognition for the overall odor of samples through specific sensors, and its detection process is real-time and non-destructive [9]. HS-SPME-GC-MS combines the high-efficiency enrichment capability of headspace solid phase microextraction, the high separation efficiency of gas chromatography and the accurate qualitative capability of mass spectrometry, and is particularly suitable for the analysis of trace volatile components in food [7]. HS-GC-IMS exhibits unique value in the non-targeted screening of trace VOCs by virtue of its advantages of rapid separation and detection at atmospheric pressure [9], which enables a more comprehensive analysis of VOCs in FG and AG. The combined use of E-nose, GC-MS, and GC-IMS effectively avoids the limitations of a single detection technology. Furthermore, OPLS-DA, cluster heatmap and rOAV analysis were adopted for further data processing, making the experimental results of this study more systematic and comprehensive.

In this study, E-nose effectively distinguished the overall odor characteristics of FG and AG, and PCA showed significant inter-group differences (Figure 1C), indicating that autoclaving had a significant impact on the flavor of fresh ginseng. Loading analysis indicated that the contents of short-chain alkanes, including methane (sensor W1S), organic sulfides (sensors W2W, W9S) and alcohols, ethers, aldehydes and ketones (sensor W8S), in AG were increased (Figure 1D). A total of 1103 VOCs were identified by GC-MS-based widely targeted metabolomics. Ten types of metabolites, including terpenoids, hydrocarbons, alcohols and aldehydes, were detected in the two groups of the samples, among which terpenoids accounted for the largest proportion, followed by esters (Figure 2B). Among the identified VOCs, 253 were significantly different metabolites, with 72 metabolites showing increased contents and 181 showing decreased contents (Figure 2). The OPLS-DA model (R2Y = 0.999, Q2 = 0.925) exhibited excellent predictive ability, indicating essential differences in flavor metabolites between the two groups of samples (Figure 4A). Furthermore, seven key compounds contributing to the overall flavor were identified by rOAV analysis (Figure 2C). Combined with the cluster heatmap and the 20 metabolites with altered contents summarized in Table 2, we concluded that certain esters such as 2(3H)-furanone, 5-heptyldihydro- (VIP = 1.24, fold change = 4.40) and some terpene derivatives increased significantly in AG, while the contents of some terpenols such as d-menthol (VIP = 1.24, fold change = 0.17) decreased (Figure 4C). This change was basically consistent with the changes in the contents of esters and terpenoids in ginseng of different ages [28], but autoclaving induced a more drastic transformation in an extremely short time in this study, which might be related to the acceleration of esterification reaction and thermal degradation of terpenoids by thermal processing [31]. In addition, HS-GC-IMS further supplemented the dynamic information of volatile components from the perspective of trace and rapid detection, and a total of 81 VOCs were qualitatively identified. The 2D difference spectrum intuitively showed that the signal intensity of AG was generally higher than that of FG group (Figure 5B). 2-furfural, phenylacetaldehyde, 3-hydroxy-2-butanone and other compounds were highly expressed in the AG group, which usually have nutty, sweet and creamy aromas. Their accumulation may be closely related to the intensification of a Maillard reaction and Strecker degradation [29]. However, the contents of terpene compounds such as α-farnesene and E-β-farnesene, which directly contribute to grassy and woody aromas in FG, were relatively reduced in AG. The changes in the types and contents of the flavor compounds transformed the overall aroma of AG from the “green and earthy” characteristics of FG to more mellow and pleasant “woody and roasted” characteristics, which was consistent with the results reported by Xiang et al. [21] that different drying methods could change the aroma type of ginseng, while the flavor enhancement effect of autoclaving was more prominent in this study.

Despite the comprehensive characterization of volatile flavor changes triggered by autoclaving, several limitations exist in this research. First, only a single autoclaving condition (135 °C, 10 min) was designed in the sample pretreatment, without setting the gradient temperature and holding time groups. We cannot quantify how processing severity regulates the conversion degree of key flavor substances, and failed to establish a quantitative correlation between thermal intensity and aroma profile transformation. Second, instrumental flavor detection was performed without collaborative human sensory panel evaluation; we could not directly link the concentration variation of volatile metabolites to human sensory descriptions (e.g., roasted note, green odor, creamy flavor). Third, this study only focused on volatile organic compounds, ignoring the interaction between non-volatile bioactive ingredients (ginsenosides, amino acids, reducing sugars) and flavor formation. The decomposition of ginsenosides and amino acid–sugar browning reactions jointly drive aroma generation during autoclaving, which requires further integrated metabolomics research combining volatile and non-volatile metabolites. For follow-up research, gradient high-temperature and high-pressure treatment parameters will be designed to build a quantitative model of processing condition–flavor quality. Human sensory evaluation combined with aroma recombination experiments will be conducted to confirm the actual sensory contribution of rOAV-screened key volatile compounds. Moreover, joint analysis of volatile and non-volatile metabolites will be carried out to reveal the full metabolic network of flavor formation in autoclaved ginseng.

## 5. Conclusions

In this study, the effects of autoclaving on volatile compounds in fresh ginseng were systematically investigated using E-nose, HS-SPME-GC-MS, and HS-GC-IMS. Autoclaving was found to exert a significant influence on the flavor profile of fresh ginseng. Notably, terpenoids, aldehydes, alcohols, and esters were identified as the major aroma components in both autoclaved ginseng and fresh ginseng. Compared with fresh ginseng, autoclaved ginseng exhibited markedly elevated contents of short-chain alkanes, organic sulfides, and aromatic compounds, which contributed to a more distinct woody aroma. This study offered theoretical support for the development and quality control of functional ginseng-based foods and health products.

## Figures and Tables

**Table 1:** Summaries for different types of sensors and their application in odor detection.

Array Serial Number	Sensor Name	Performance Description
1	W1C	Selective for benzene and other aromatic hydrocarbons
2	W5S	Highly sensitivity to nitrogen oxides; responsive to acidic nitrogen containing volatiles
3	W3C	Dual response to ammonia and aromatic volatile compounds
4	W6S	Specifically sensitive to hydrogen gas
5	W5C	Detects aliphatic hydrocarbons and low-polarity aromatic substances
6	W1S	High sensitivity toward short-chain alkanes including methane
7	W1W	Strong response to inorganic sulfide volatiles
8	W2S	Broad-spectrum response to alcohols, ethers, aldehydes and ketones
9	W2W	Captures aromatic compounds and organosulfur volatile metabolites
10	W3S	Predominantly responsive to long-chain aliphatic alkanes

**Table 2:** Top 20 key differential VOCs between FG and AG.

Compounds	Class	FG Mean ± SD	AG Mean ± SD	VIP	*p*-Value	FDR	Fold_Change	Type
2(3H)-furanone, 5-heptyldihydro-	Ester	7700.71 ± 252.31 ^b^	33,859.69 ± 1552.82 ^a^	1.24015252301346	0.000905771538461467	0.0102832211345575	4.3969585830533	up
Benzoic acid, 2-(acetylamino)-, methyl ester	Ester	40,273.30 ± 536.84 ^b^	93,925.27 ± 1683.99 ^a^	1.24002003417583	0.0000978276507603363	0.00372082409616038	2.33219689800704	up
Hexanoic acid, octyl ester	Ester	1950.88 ± 90.01 ^b^	5777.53 ± 37.17 ^a^	1.23948600258623	0.0000216093871690843	0.00129339627804877	2.96150031012624	up
2-naphthalenemethanol, 2,3,4,4a,5,6,7,8-octahydro-.alpha.,.alpha.,4a,8-tetramethyl-, [2R-(2.alpha.,4a.beta.,8.beta.)]-	Terpenoids	72,572.26 ± 2385.90 ^b^	188,827.83 ± 3582.05 ^a^	1.23883189786795	0.0000053249196920098	0.000870180201264822	2.60192836823226	up
Ketone, methyl 2,4,5-trimethylpyrrol-3-yl	Ketone	1362.07 ± 86.01 ^b^	12,112.91 ± 591.13 ^a^	1.23856214951201	0.000812450707610136	0.00998414240659764	8.89304348088472	up
2-methyl-6-(p-tolyl)hept-2-en-4-ol	Terpenoids	10,026.40 ± 385.77 ^b^	20,195.68 ± 440.16 ^a^	1.2385518700623	8.50120527376671 × ^−06^	0.0010418699352183	2.01425127989296	up
N-nenzyloxy-2-carbomethoxyaziridine	Heterocyclic compound	35,065.36 ± 1184.86 ^b^	77,027.79 ± 1679.62 ^a^	1.23815810518893	0.0000105658006790846	0.00105946164991185	2.19669167751018	up
(1R,3E,7E,11R)-1,5,5,8-tetramethyl-12-oxabicyclo [9.1.0]dodeca-3,7-diene	Terpenoids	21,098.13 ± 998.18 ^b^	55,551.44 ± 328.99 ^a^	1.23808276696333	0.0000744460443820918	0.00335217197400903	2.63300292120642	up
Diethyltoluamide	Amine	26,190.99 ± 823.99 ^b^	58,661.79 ± 2546.09 ^a^	1.23723391903237	0.000857855644746286	0.0101743524317758	2.23977010096259	up
2-n-heptylfuran	Heterocyclic compound	3085.26 ± 161.39 ^b^	18,007.42 ± 1911.18 ^a^	1.23702377716495	0.00516602879336966	0.0261382099040676	5.83659146612067	up
d-menthol	Terpenoids	69,377.27 ± 3051.73 ^a^	12,024.69 ± 0.00 ^b^	1.24138389774367	0.000942431069989984	0.0102832211345575	0.173323194363126	down
Cyclohexanol, 5-methyl-2-(1-methylethyl)-, (1.alpha.,2.alpha.,5.beta.)-	Terpenoids	69,377.27 ± 3051.73 ^a^	12,024.69 ± 0.00 ^b^	1.24138389774367	0.000942431069989984	0.0102832211345575	0.173323194363126	down
Furyl hydroxymethyl ketone	Ketone	11,544.12 ± 694.21 ^a^	1049.17 ± 0.00 ^b^	1.24016584632548	0.001455291032797	0.0118898527270508	0.0908838054322469	down
Carotol	Terpenoids	1,169,589.64 ± 44,446.09 ^a^	271,846.38 ± 1077.56 ^b^	1.24010204821239	0.000810963621162957	0.00998414240659764	0.232428855165435	down
cis-3-hexenylpyruvate	Ester	6360.15 ± 170.16 ^a^	1960.46 ± 117.74 ^b^	1.23947902738482	0.0000100643866195569	0.00105946164991185	0.30824165498399	down
7-epi-a-eudesmol	Terpenoids	36,088.23 ± 1704.47 ^a^	17,861.71 ± 594.73 ^b^	1.23863470194252	0.00116370651439862	0.0110652438394973	0.494945649733258	down
2,6,6-trimethyl-2-cyclohexene-1,4-dione	Ketone	8961.56 ± 566.41 ^a^	2777.08 ± 129.67 ^b^	1.23852355025785	0.00183665986458793	0.0136880799367601	0.309887744252954	down
1-decen-3-one	Ketone	13,063.86 ± 651.27 ^a^	4190.64 ± 223.96 ^b^	1.23816628267572	0.000657722518031928	0.0095729303561331	0.320781470615889	down
Benzeneacetic acid, .alpha.-cyclopentyl	Acid	11,132.62 ± 949.88 ^a^	1768.59 ± 211.05 ^b^	1.23765348320266	0.00235063022388085	0.0170575337956617	0.15886561155353	down
Benzoic acid, 2-(methylamino)-, 2-methylpropyl ester	Ester	53,736.75 ± 2358.24 ^a^	21,969.09 ± 1281.95 ^b^	1.23754160234007	0.000211248494042513	0.00519318991688959	0.408828132777272	down

Note: Data are mean ± standard deviation (*n* = 3); data in the same column with different superscripts are significantly different (*p* < 0.05). Table 2 is a truncated summary of top 20 key differential VOCs extracted from Supplementary Table S1. Full identification evidence, including EI mass spectra, fragment ions and library matching scores for all differential volatile metabolites, is provided in Table S1 (Supplementary Materials).

**Table 3:** Determination of volatile flavor compounds in FG and AG by GC-IMS.

No.	Compound	CAS#	Formula	MW	RI	Rt (s)	Dt (RIPrel)
1	beta-damascone	C35044689	C_13_H_20_O	192.3	1955.5	3351.594	1.43763
2	delta-cadinene	C483761	C_15_H_24_	204.4	1907.2	3019.644	1.47595
3	alpha-farnesene-M	C502614	C_15_H_24_	204.4	1865.4	2758.438	1.43911
4	alpha-farnesene-D	C502614	C_15_H_24_	204.4	1866.7	2766.6	1.49363
5	phenylacetaldehyde	C122781	C_8_H_8_O	120.2	1781.0	2298.605	1.25785
6	(E)-beta-farnesene-M	C18794848	C_15_H_24_	204.4	1771.7	2253.029	1.44141
7	(E)-beta-farnesene-D	C18794848	C_15_H_24_	204.4	1772.2	2255.443	1.49092
8	(Z)-beta-farnesene-M	C28973979	C_15_H_24_	204.4	1739.3	2100.927	1.43506
9	(Z)-beta-farnesene-D	C28973979	C_15_H_24_	204.4	1739.9	2103.341	1.49092
10	beta-cyclocitral	C432257	C_10_H_16_O	152.2	1704.6	1948.825	1.32462
11	(E)-caryophyllene	C87445	C_15_H_24_	204.4	1698.2	1922.267	1.4376
12	(E,E)-2,4-octadienal	C30361285	C_8_H_12_O	124.2	1695.3	1910.196	1.26622
13	propanoic acid	C79094	C_3_H_6_O_2_	74.1	1647.3	1721.879	1.11135
14	2-undecanone	C112129	C_11_H_22_O	170.3	1669.8	1807.745	1.53532
15	1-octanol	C111875	C_8_H_18_O	130.2	1669.4	1806.155	1.47373
16	beta-elemene	C515139	C_15_H_24_	204.4	1657.4	1760.035	1.43992
17	(E)-2-nonenal-M	C18829566	C_9_H_16_O	140.2	1586.1	1508.763	1.41456
18	ethyl nonanoate	C123295	C_11_H_22_O_2_	186.3	1587.6	1513.534	1.52687
19	(E)-2-nonenal-D	C18829566	C_9_H_16_O	140.2	1586.6	1510.353	1.97129
20	2-decanone	C693549	C_10_H_20_O	156.3	1564.6	1440.378	1.47977
21	benzaldehyde	C100527	C_7_H_6_O	106.1	1561.1	1429.424	1.15533
22	2,3-diethyl-5-methylpyrazine	C18138040	C_9_H_14_N_2_	150.2	1528.7	1332.836	1.26617
23	acetic acid-M	C64197	C_2_H_4_O_2_	60.1	1511.4	1283.959	1.05143
24	acetic acid-D	C64197	C_2_H_4_O_2_	60.1	1511.9	1285.123	1.15418
25	2-furfural-M	C98011	C_5_H_4_O_2_	96.1	1503.8	1263.013	1.08606
26	2-furfural-D	C98011	C_5_H_4_O_2_	96.1	1504.2	1264.176	1.33313
27	(E,E)-2,4-heptadienal	C4313035	C_7_H_10_O	110.2	1526.3	1325.853	1.19805
28	1-octen-3-ol	C3391864	C_8_H_16_O	128.2	1497.4	1245.557	1.16572
29	citronellal	C106230	C_10_H_18_O	154.3	1504.2	1264.176	1.20959
30	2-ethyl-1-hexanol	C104767	C_8_H_18_O	130.2	1499.5	1251.375	1.40586
31	(Z)-3-hexenyl isovalerate	C35154451	C_11_H_20_O_2_	184.3	1500.8	1254.867	1.47282
32	linalool oxide	C60047178	C_10_H_18_O_2_	170.3	1459.1	1146.641	1.25693
33	(E)-2-octenal-M	C2548870	C_8_H_14_O	126.2	1448.2	1119.876	1.33775
34	(E)-2-octenal-D	C2548870	C_8_H_14_O	126.2	1449.1	1122.203	1.82149
35	2,4-hexadienal	C142836	C_6_H_8_O	96.1	1441.9	1104.747	1.4405
36	tetrahydrolinalool	C78693	C_10_H_22_O	158.3	1428.5	1073.327	1.26732
37	dimethyl trisulfide	C3658808	C_2_H_6_S_3_	126.3	1426.5	1068.672	1.3008
38	nonanal	C124196	C_9_H_18_O	142.2	1413.2	1038.415	1.47744
39	2-Nonanone	C821556	C_9_H_18_O	142.2	1409.6	1030.269	1.41048
40	methyl octanoate	C111115	C_9_H_18_O_2_	158.2	1413.2	1038.415	1.43703
41	1-hexanol-M	C111273	C_6_H_14_O	102.2	1379.3	965.101	1.33197
42	1-hexanol-D	C111273	C_6_H_14_O	102.2	1379.9	966.265	1.64023
43	6-methyl-5-hepten-2-one	C110930	C_8_H_14_O	126.2	1367.2	940.144	1.17095
44	ethyl lactate	C97643	C_5_H_10_O_3_	118.1	1357.1	919.874	1.1335
45	(E)-2-heptenal-M	C18829555	C_7_H_12_O	112.2	1339.8	886.09	1.26128
46	(E)-2-heptenal-D	C18829555	C_7_H_12_O	112.2	1339.8	886.09	1.67215
47	cis-2-penten-1-ol	C1576950	C_5_H_10_O	86.1	1347.6	901.15	0.94765
48	3-hydroxy-2-butanone-M	C513860	C_4_H_8_O_2_	88.1	1308.1	827.485	1.06194
49	3-hydroxy-2-butanone-D	C513860	C_4_H_8_O_2_	88.1	1307.7	826.784	1.33149
50	octanal-M	C124130	C_8_H_16_O	128.2	1311.2	833.098	1.40372
51	octanal-D	C124130	C_8_H_16_O	128.2	1310.9	832.396	1.8296
52	1-pentanol-M	C71410	C_5_H_12_O	88.1	1274.4	771.36	1.25817
53	1-pentanol-D	C71410	C_5_H_12_O	88.1	1274.7	771.757	1.5135
54	3-octanone	C106683	C_8_H_16_O	128.2	1267.5	760.523	1.3055
55	2-pentylfuran	C3777693	C_9_H_14_O	138.2	1252.4	737.521	1.25813
56	(E)-2-hexenal-M	C6728263	C_6_H_10_O	98.1	1241.6	721.472	1.18605
57	(E)-2-hexenal-D	C6728263	C_6_H_10_O	98.1	1241.2	720.937	1.5238
58	3-methyl-1-butanol-M	C123513	C_5_H_12_O	88.1	1230.6	705.424	1.24887
59	3-methyl-1-butanol-D	C123513	C_5_H_12_O	88.1	1230.9	705.959	1.49085
60	3-methyl-2-butenal-M	C107868	C_5_H_8_O	84.1	1224.2	696.329	1.09441
61	3-methyl-2-butenal-D	C107868	C_5_H_8_O	84.1	1224.6	696.864	1.35905
62	pyridine	C110861	C_5_H_5_N	79.1	1205.8	670.652	1.24578
63	heptanal-M	C111717	C_7_H_14_O	114.2	1206.9	672.257	1.3333
64	heptanal-D	C111717	C_7_H_14_O	114.2	1207.3	672.792	1.69988
65	1-penten-3-ol	C616251	C_5_H_10_O	86.1	1183.9	634.275	0.9451
66	1-butanol-M	C71363	C_4_H_10_O	74.1	1170.3	605.388	1.18605
67	1-butanol-D	C71363	C_4_H_10_O	74.1	1170.6	605.923	1.3817
68	3-penten-2-one	C625332	C_5_H_8_O	84.1	1155.5	575.431	1.33536
69	isoamyl acetate	C123922	C_7_H_14_O_2_	130.2	1146.4	557.777	1.74416
70	butyl propanoate	C590012	C_7_H_14_O_2_	130.2	1144.4	554.033	1.29314
71	3-carene	C13466789	C_10_H_16_	136.2	1141.0	547.613	1.21797
72	alpha-phellandrene	C99832	C_10_H_16_	136.2	1182.4	631.065	1.219
73	limonene	C138863	C_10_H_16_	136.2	1224.6	696.864	1.21386
74	beta-pinene-M	C127913	C_10_H_16_	136.2	1126.4	520.866	1.21797
75	beta-pinene-D	C127913	C_10_H_16_	136.2	1126.1	520.331	1.64119
76	2-methyl-1-propanol-M	C78831	C_4_H_10_O	74.1	1119.4	508.562	1.17679
77	2-methyl-1-propanol-D	C78831	C_4_H_10_O	74.1	1120.1	509.632	1.3714
78	hexanal	C66251	C_6_H_12_O	100.2	1111.0	494.118	1.56808
79	ethyl 3-methylbutanoate	C108645	C_7_H_14_O_2_	130.2	1092.4	464.696	1.28182
80	dimethyl disulfide	C624920	C_2_H_6_S_2_	94.2	1097.1	471.115	1.1531
81	camphene	C79925	C_10_H_16_	136.2	1086.4	456.791	1.21959
82	(E)-2-butenal	C123739	C_4_H_6_O	70.1	1075.1	442.034	1.20131
83	1-propanol	C71238	C_3_H_8_O	60.1	1067.3	432.307	1.24749
84	alpha-pinene	C80568	C_10_H_16_	136.2	1048.0	408.829	1.6651
85	1-penten-3-one	C1629589	C_5_H_8_O	84.1	1053.1	414.866	1.31389
86	2-butanol	C78922	C_4_H_10_O	74.1	1045.4	405.811	1.32062
87	2-ethylbutanal	C97961	C_6_H_12_O	100.2	1027.8	385.687	1.50826
88	2-pentanone	C107879	C_5_H_10_O	86.1	1010.8	367.24	1.36585
89	ethyl propanoate	C105373	C_5_H_10_O_2_	102.1	987.9	345.774	1.45533
90	2-ethylfuran	C3208160	C_6_H_8_O	96.1	968.0	331.016	1.31004
91	ethanol	C64175	C_2_H_6_O	46.1	951.9	319.613	1.13587
92	acetal	C105577	C_6_H_14_O_2_	118.2	919.0	297.476	1.03773
93	2-methyl-2-propanol	C75650	C_4_H_10_O	74.1	934.3	307.538	1.32447
94	2-butanone	C78933	C_4_H_8_O	72.1	930.3	304.855	1.25038
95	ethyl acetate	C141786	C_4_H_8_O_2_	88.1	908.6	290.768	1.3389
96	acetone	C67641	C_3_H_6_O	58.1	845.8	253.539	1.11759
97	methyl acetate	C79209	C_3_H_6_O_2_	74.1	864.3	263.936	1.19649
98	propanal-M	C123386	C_3_H_6_O	58.1	831.7	245.825	1.06948
99	propanal-D	C123386	C_3_H_6_O	58.1	831.7	245.825	1.14646
100	acetaldehyde	C75070	C_2_H_4_O	44.1	778.1	218.657	1.02137
101	3-methylbutanal	C590863	C_5_H_10_O	86.1	943.2	313.576	1.41492

Note: M = monomer ion peak of the compound; D = dimer ion peak of the compound; Dt (RIPrel) = relative drift time normalized to the reaction ion peak (RIP); RI = retention index; Rt = gas chromatographic retention time (s); CAS# = Chemical Abstracts Service registry number; MW = molecular weight.

## Data Availability

The original contributions presented in the study are included in the article/Supplementary Materials. Further inquiries can be directed to the corresponding author.

## Supplementary Materials

The following supporting information can be downloaded at: https://www.mdpi.com/article/10.3390/foods15183289/s1, Table S1: Full dataset of differential volatile organic compounds between fresh ginseng (FG) and autoclaved ginseng (AG).
