# Supplementary material for: Analysis of the Effect of Autoclaving on the Flavor of Fresh Ginseng Using Electronic Nose, HS-GC-IMS, and HS-SPME-GC-MS
Source: Foods. 2026 Sep 17;15(18):3289. doi: 10.3390/foods15183289 (PMC13606668; doi:10.3390/foods15183289)
Supplement: Supplementary file 1 [file foods-15-03289-s001.zip › foods-4449493-supplementary.pdf]

Table S1. Full dataset of differential volatile organic compounds between fresh ginseng (FG) and autoclaved ginseng (AG)

Index	Quantitative ion	Qualitative ion	Molecular weight (Da)	Compounds	Class I	CAS	NIST_RI	Odor	Threshold	cpd_ID	FG1	FG2	FG3	AG1	AG2	AG3	QC01	QC02	QC03	AG_vs_FG_VIP	AG_vs_FG_P-value	AG_vs_FG_Fold_Change	Type
WAMW0630*017	68	93	136.125	Cyclohexene, 1-methyl-5-(1-methylethenyl)-, (R)-	Terpenoids	1461-27-4	1027	-	0.045	-	48462.9633620151	41052.3093520206	32331.6857683717	11871.9174226074	9869.13203628545	8579.82963395692	26241.7432005769	26082.2092056886	25181.4209231874	1.208908914	0.019122	0.248844	down
QWMW0297*095	71	81	156.151	d-Menthol	Terpenoids	15356-60-2	1167	caraway, sweet	0.92	-	65893.998031876	71580.5728064581	70657.245008759	12024.69039040536	12024.69039040536	12024.69039040536	64014.7651022954	60123.4519520268	61429.5066320195	1.241383898	0.000942	0.173323	down
NMW0791*218	105	120	120.094	Benzene, 1-ethyl-2-methyl-	Aromatics	611-14-3	971	-	0.36	C14572	14896.3236640011	13397.4172313229	10634.49990484459	1626.20000357662	1626.20000357662	1626.20000357662	8843.62953283672	8131.0000178831	8561.14392575054	1.23435	0.011879	0.125323	down

																				539			
NMW0783*011	93	91	136.125	(1S)-2,6,6-Trimethylbicyclo[3.1.1]hept-2-ene	Terpenoids	7785-26-4	936.524453694069	sharp, warm, resinous, fresh, pine	0.1	C06308	800422.055898131	744229.399208525	493300.796847228	232768.349228149	209861.294824462	172751.408988113	493071.907169844	479857.114023376	472514.67356668	1.187839289	0.033749	0.30196	down
NMW0310*459	69	107	222.198	2,6,10-Dodecatrien-1-ol, 3,7,11-trimethyl-	Terpenoids	4602-84-0	1702.6373626373 6	mild, fresh, sweet, linden, floral, angelica	1	C01493	5335.20891192646	5035.564240600 82	4505.325720459 17	2480.942579711 99	2615.210440918 05	2216.879643737 88	3392.11154095473	4035.67044458022	4150.41604638687	1.215313658	0.003034	0.491596	down
NMW0272*318	127	115	173.048	Naphthalene, 2-nitro-	Nitrogen compounds	581-89-5	1638	-	-	C19474	161307.696251872	176079.883867226	184446.179364052	53869.3890836425	63406.794140038	61299.94294310 83	100575.399770068	109260.990817858	109372.00748018	1.234878258	0.000999	0.342209	down
NMW0271*302	118	164	164.047	p-Coumaric acid	Acid	7400-08-0	1635	-	23	-	99028.1271521488	105205.478233507	110935.52597307	37471.1498222617	45027.203061885	43086.11657787 64	68715.3738884198	70824.4415760416	67755.63080663 28	1.2314	0.000263	0.398467	down

NMW0227*137	93	119	204.188	(Z)-1-Methyl-4-(6-methylhept-5-en-2-ylidene)cyclohex-1-ene	Terpenoids	13062-00-5	1515	-	-	-	798413.262021469	787644.091966049	666109.812415515	312413.274434564	302920.58750268	282953.520335932	497461.537589348	526587.123727041	509735.035969716	184311.227705919	0.006856	0.398855	down
WAMW2219*199	41	68	112.089	4-Heptenal, (E)-	Aldehyde	929-22-6	905	-	0.034	-	4315.64540960693	3923.95670809936	3456.07794714354	13551.2488166504	12974.2455733643	14282.5470191651	7481.32861962891	6234.18448602295	6686.2235975647	1.234416059	8.51E-05	3.489155	up
WAMW1799*181	55	41	100.052	2-Pentenoic acid	Acid	626-98-2	873	pungent, fatty	-	-	19905.892184204	21324.6982518005	36425.8605141297	4088.58513793946	5565.31051229859	3263.27225018309	14085.7504959716	12390.0418035278	12364.3120760193	1.195886222	0.052987	0.166337	down
WAMW1749	105	120	120.094	Benzene, 1-ethyl-4-methyl-	Aromatics	622-96-8	954	-	0.041	-	12740.2891000211	10360.7668805845	7497.57771084583	1240.29215329588	1240.29215329588	1240.29215329588	6493.36911451711	6371.21609757985	6201.4607664794	1.22	0.027444	0.121603	down

*218																				5785032			
WAMW1632*198	69	56	112.089	Cyclohexanone, 3-methyl-	Ketone	591-24-2	958	minty, camphor, medicinal	0.46	-	3806.34166540525	3493.85394396968	7249.75169033811	13209.4958898619	11877.6597295226	13557.9709316252	7328.76979844665	7872.18545567318	7234.81593630981	1.132009187	0.011701	2.656032	up
WAMW0477*112	112	69	154.136	Cyclohexanone, 5-methyl-2-(1-methylethyl)-, (2R-cis)-	Ketone	1196-31-2	1157	minty	0.15	C11952	81952.1773399051	85370.5926334843	55835.4316630251	183925.57988672	181735.192689088	198027.66899141	146754.631098328	141377.652801819	146374.44499762	1.189455375	0.001518	2.525959	up
QWMW1295*211	45	55	116.12	2-Heptanol, (S)-	Alcohol	6033-23-4	903	mushroom	-	-	2739.51089807127	2762.64667807007	2653.09248400878	6960.46241250613	7094.1097773743	7852.30238706973	4562.07834967041	4295.17425628662	3955.81262411499	1.235984325	0.003273	2.68623	up
KMW0	204	161	204.188	Naphthalene, 1,2,3,4,4a,5,6	Terpenoids	39029-41-9	1513	herbal, woody	-	-	179097.14837	174573.4532	148088.680	85478.71197	80945.4111	78779.8023	121171.46	125874.69	119805.912	1.	0.01017	0.488688	down

616*152				,8a-octahydro-7-methyl-4-methylene-1-(1-methylethyl)-, (1.alpha.,4a.beta.,8a.alpha.)-							537	59769	34949	02161	945812	974622	5667971	5066408	268068	219998051	3		
NMW0075*093	93	121	154.136	Cyclohexanol, 1-methyl-4-(1-methylethylidene)-	Terpenoids	586-81-2	1197	terpineol, lilac	-	C17518	2495.7618508148	2439.60077786251	2088.84968572995	6725.57979083238	7800.96759994485	6182.26023985279	4781.39365234367	5554.52130232231	4865.3523726195	1.225601724	0.007362	2.948204	up
NMW0065*092	95	110	154.136	Bicyclo[2.2.1]heptan-2-ol, 1,7,7-trimethyl-, (1S-endo)-	Terpenoids	464-45-9	1170	pine, woody, camphor	0.048	C01766	188148.340954591	196957.63712964	132272.609931214	420459.531877504	413035.676212282	450059.618242495	334984.835125429	324374.154021791	330198.789915255	1.19377539	0.001308	2.480881	up
NMW0034*400	80	69	152.12	Fenchone	Terpenoids	1195-79-5	1096	herbal, cedar leaf, bitter, thuja, camphor, earthy, woody	2.3	-	17693.9931960599	17568.4264842527	17389.9714000241	2393.9686499023	2545.89336996454	478.79372998046	9627.85353552231	10164.8200393522	9046.96216506945	1.133300692	0.00152	0.102914	down
KM	111	123	222.19836	β-CARYOP	Terpenoids	472-97	1568	warm,	-	-	1894.0	2617.9	2779.	23474.	28240	27912	1339	1290	12030	1	0.00	10.920	up

W0650*382			5	HYLLENE ALCOHOL		-9		woody, moss, spicy, earthy			0623474118	2797650146	8048170166	5361446531	.47205749 49	.58518957 49	8.8031282958	0.4769909056	.23193310 54	.230254972	3211	25	
KMW0644*118	159	177	220.183	1H-Cyclopro p[e]azulen-7-ol, decahydro-1,1,7-trimethyl-4-methylene-, [1ar-(1a.alpha.,4a.alpha.,7.beta.,7a.beta.,7b.alpha.)]-	Terpenoids	6750-60-3	1576	earthy, herbal, fruity	-	-	17418.8947900998	17034.2697509763	17183.35907482 89	8290.79133935537	7748.0463397215 9	9109.6375382078 9	11832.2619730223	13262.7976318968	12814.93792456 03	1.230681951	0.001018	0.487029	down
WMW0018*380	119	41	222.198	.tau.-Cadinol	Terpenoids	5937-11-1	1640	pure, balsamic, earthy	-	-	1217037.36168759	1338067.13994465	1389052.39599494	431818.008150341	496250.34465375 2	472102.80783930 1	779078.137346339	834948.561939107	829325.06819423 4	1.236045424	0.001389	0.354999	down
XMW1403*042	82	67	184.146	cis-3-Hexenyl isovalerate	Ester	35154-45-1	1238	fresh, green, apple, fruity, tropical, pineapple	0.02	-	1345.16392333981	1610.00808883663	269.03278466796 2	6500.40905203238	6753.6689966735	6659.2932384184 6	4623.15826757805	4133.24018054192	4053.24080776971	1.08572130	0.004332	6.176212	up

																				6			
XMW1170*142	136	121	238.193	Butanoic acid, 2-methyl-, 3,7-dimethyl-2,6-octadienyl ester, (Z)-	Ester	51117-19-2	1576	-	-	-	7781.81138055458	9184.51130798377	7919.70201367217	18795.6763169871	17421.71431317233	17231.38595959554	12273.4917277227	12662.0143253178	11847.27236917177	1.222884967	0.000147	2.147743	up
XMW1069*313	67	82	170.131	Butanoic acid, 3-hexenyl ester, (E)-	Ester	53398-84-8	1185	-	-	-	6984.82780780033	6638.72054156496	6994.663201843299	2511.9228215332	2285.124359863277	2948.458842224133	5555.31633880618	5138.43358398438	5054.933760772771	1.226375464	0.000185	0.375663	down
XMW0967*361	136	121	204.188	(1R,5S)-1,8-Dimethyl-4-(propan-2-ylidene)spiro[4.5]dec-7-ene	Terpenoids	28400-12-6	1515	-	-	-	43036.4350910642	42748.9412239988	36546.32123925744	14801.7596207274	14642.55783703611	13730.139649536	26340.609210022	28261.2364161984	27338.94590441866	1.232718734	0.005435	0.352929	down
XMW0935*447	150	135	150.104	(E)-2,6-Dimethylocta-2,5,7-trien-4-one	Terpenoids	33746-72-4	1239	-	-	-	118262.37671388	111366.516603086	93608.78678806888	34385.2358811334	31939.12171189822	27131.19688172866	67487.6549403826	68061.8603887162	67319.98952030811	1.223284	0.005717	0.289123	down

XMW0902*261	69	41	138.141	2,6-Dimethyl-2-trans-6-octadiene	Terpenoids	2609-23-6	993	-	-	-	40120.1400521238	41377.4183155515	26950.91271984844	12442.2581413268	9504.225996673544	10025.79207708731	25619.7656520384	29856.5257326047	22763.41196667466	1621.198856364	0.027471	0.294815	down
XMW0890*228	108	58	126.104	1-Hepten-6-one, 2-methyl-	Ketone	10408-15-8	966	-	-	-	9329.98769398477	8274.87495941142	6863.806824294888	2369.71764199822	473.94352839964404	473.94352839964404	5155.36611352525	4926.41958952319	5440.808369811877	1.116438678	0.001892	0.135586	down
XMW0853*379	135	81	222.198	Neointermedeol	Terpenoids	5945-72-2	1660	-	-	-	23038.2793085321	20963.5114210199	22034.99536035099	7821.03299462869	7535.645125488055	8422.069401305922	12418.1585792843	14067.4652895504	11908.83718505822	1.235345984	0.000392	0.360083	down
w09*261	69	41	138.141	2,6-Octadiene, 2,6-dimethyl-	Terpenoids	2792-39-4	978	-	-	-	113774.444750058	104041.971725034	75024.84398913399	27807.7608296807	20400.07447842355	19300.03951385455	60615.3401866439	65260.2617872909	61892.29271337744	1.2060	0.018947	0.230527	down

																				63209			
YZMW0343*254	51	93	136.089	Phenol, 2,4,5-trimethyl-	Phenol	496-78-6	1234	-	2.3	-	8851.89920005773	8174.38603442357	6896.334587798855	2769.08507675162	2343.257523345888	2230.510276138244	5556.19422334274	5212.83944894396	5098.241671356077	1.2215290351	0.006927	0.306942	down
YZMW0320*015	79	121	136.125	Bicyclo[4.1.0]hept-2-ene, 3,7,7-trimethyl-, (1S-cis)-	Terpenoids	4497-92-1	985	-	4	-	11487.4166761473	12633.6235126341	9801.056311706466	3863.90040240473	2889.456827148422	3127.959033203099	8238.74503100576	7144.36733544915	7825.445639160077	1.2207581121	0.005481	0.291294	down
XMW0234*366	119	93	204.188	cis-.alpha.-Bergamotene	Terpenoids	18252-46-5	1415	-	-	-	276837.143825684	272510.10469922	224321.278349367	102858.459043939	93269.4935256297	88341.4606350049	178996.587872072	185764.179780031	180726.644820314	1.2237138071	0.00747	0.367689	down
XMW0187*02	82	71	152.12	1,5,7-Octatrien-3-ol, 3,7-dimethyl-	Alcohol	29957-43-5	1107	mouldy	0.11	-	4761.94916809083	6646.09709020999	5232.977543701199	12977.3273486329	14382.08769238299	9551.699337341388	11537.7555530396	9681.14264636237	9920.330291137755	1.14	0.029229	2.21808	up

4																				7250794			
XMW0009*064	189	161	204.188	Naphthalene, 1,2,4a,5,8,8a-hexahydro-4,7-dimethyl-1-(1-methylethyl)-, [1S-(1.alpha.,4a.beta.,8a.alpha.)]-	Terpenoids	523-47-7	1518	green, woody	-	C09625	566681.342471687	548975.284249031	469131.018474371	225457.92882654	220209.992075075	212681.360136721	355074.379186161	372384.375910649	362740.796031622	1.228135316	0.008529	0.415418	down
XMW0004*119	41	93	136.125	Bicyclo[3.1.1]heptane, 6,6-dimethyl-2-methylene-, (1S)-	Terpenoids	18172-67-3	979.707876890976	dry, woody, fresh, pine, hay, green, resinous	4.16	C06307	39601.1848816518	36995.6447611074	27771.8697696526	8995.54989773534	7455.06979608133	5637.92468554671	21717.5649625238	22229.9415914301	20507.932061156695	1.208432098	0.012136	0.21164	down
WMW0215*018	93	91	136.125	.beta.-Ocimene	Terpenoids	13877-91-3	1037	apple, pear, fruity	0.034	-	46268.845510147	40248.3136492005	30387.746502655	11768.8962056579	9412.48510003655	8245.08597807309	25212.1105211486	24858.3189111785	23780.6432929076	1.204528272	0.020125	0.251713	down
WM	70	41	138.104	2,6-Nonadienal, (E,E)-	Aldehyde	17587-33-6	1153	fresh, citrus,	0.0005	-	21064.780243	20542.85950	19894.8598	6603.016080	6977.31862	6186.33728	14233.370	15281.037	14885.2997	1.	1.5E-0	0.321396	down

W0094*032								green, cucumber, melon			4082	93994	374634	2918	081915	698738	4739381	9852295	673341	237342588	5		
XMW0312*006	91	106	106.078	Ethylbenzene	Aromatics	100-41-4	862.671719564188	-	2.20525	C07111	13085.7395057373	14006.2112203369	24086.54921826 17	4879.75911813355	4335.310103881 83	4726.989482666 02	9381.38698034 668	11329.5919867 554	12902.878910247 8	1.1855258	0.07169	0.27242	down
KMW0447*285	93	69	154.136	3,6-Octadien-1-ol, 3,7-dimethyl-, (Z)-	Terpenoids	5944-20-7	1240	-	-	-	14414.4097798305	13223.7075906063	10600.237887695	5120.95969131459	4907.689273132 21	4221.552413085 83	8523.43434260 538	8878.05979618 814	8737.655373412 87	1.208794528	0.015156	0.372668	down
D310*359	187	159	202.172	(8R,8aS)-8,8a-Dimethyl-2-(propan-2-ylidene)-1,2,3,7,8,8a-hexahydronaphthalene	Terpenoids	27840-40-0	1540	-	-	-	20459.8230146483	23199.935709778	19529.385385498 2	9705.43296374512	8970.194004028 36	8132.567005615 24	16123.6810369 873	15055.8082293 701	15384.443326172	1.225129743	0.003327	0.424253	down
D314*0	109	82	226.193	8-Dodecen-1-ol, acetate,	Ester	28079-04-1	1605	-	-	-	49514.929727	48633.37785	43532.5192	108434.0421	111817.716	106167.069	82525.731	84417.034	81505.6160	1.	1.8E-0	2.303903	up

67				(Z)-							7819	8519	370592	18527	706905	566158	1470922	1856662	483374	23495505	5		
KMW0280*139	95	43	124.089	3,5-Octadien-2-one	Ketone	38284-27-4	1091	fruity, fatty, mushroom	-	-	35874.9083925012	37055.984670318	35410.0083753962	1010.7971204284 48	5053.985602142 24	1010.797120428448	19069.7013900753	18777.4092108761	19231.2039881283	1.170530193	0.000509	0.065308	down
GMW0395*056	161	105	204.188	(3R,3aR,7R,8aS)-3,8,8-Trimethyl-6-methyleneoctahydro-1H-3a,7-methanoazulene	Terpenoids	79120-98-2	1414	-	-	-	226953.464537096	228041.830611803	181901.990045155	103269.644876703	77567.6811437938	90470.3439706981	149166.999439444	154412.055249502	150771.366836661	1.200039652	0.006157	0.425983	down
KMW0326*263	55	70	140.12	2-Nonenal, (Z)-	Aldehyde	60784-31-8	1148	orris, fatty, waxy, cucumber	0.0045	-	18230.515453064	19083.3957853393	17292.5926511231	6179.70374179081 05	6589.547097900 43	5728.456563476 62	14313.5585128174	13467.3359269409	13518.2281118774	1.236263094	0.000308	0.338745	down
D26	70	85	172.146	Pentanoic	Ester	2050-0	1108	fruity,	0.1	-	9098.4	8694.3	7646.	15684.	20096	15593	1553	1547	15793	1	0.02	2.0195	up

*145				acid, 3-methylbutyl ester		9-1		ripe apple, green			7357666019	7298919683	04262811282	2280886537	.34533007833	.35267669 68	8.2391706544	8.2283851319	.08829397599	.19540691	1334	04	
KMW0156*012	41	83	112.089	2-Heptenal, (Z)-	Aldehyde	57266-86-1	958	-	0.013	-	3628.85362329103	4879.37319482424	3380.171697814 96	9593.44851898187	9027.258952941 81	9664.393996505 68	6185.27920471189	6326.87885449215	6529.18041366574	1.194180658	0.002558	2.379219	up
D290*080	133	105	148.089	2-Butanone, 4-phenyl-	Ketone	2550-26-7	1236	floral, balsamic	2.5	-	24889.7815196527	25172.8040430598	19634.930598022	8072.25610342393	7171.7555282286	6715.539608840 82	16110.9029293057	15079.0560270382	14500.3088358456	1.222863139	0.009832	0.315069	down
D85*342	103	57	186.162	Butanoic acid, 3-methyl-, hexyl ester	Ester	10032-13-0	1244	sweet, green fruit, apple, unripe apple skin, strawberry	-	-	22773.8852793725	21872.4943322443	18276.256131347 2	6472.5442756041	6785.503152038 43	5088.810990173 21	13099.1921672512	13262.3991186672	13074.26706033 29	1.219278751	0.003917	0.291578	down

KMW0439*285	93	69	154.136	2,6-Octadien-1-ol, 3,7-dimethyl-, (Z)-	Terpenoids	106-25-2	1230.47464940669	lemon, fresh	0.049	C09871	13068.6376558684	11379.8937100829	9039.5741423644	5237.7653427734	4748.0588760986	3889.4428555907 9	8049.08318936153	8300.78902827448	7722.5107173766 6	1.178202549	0.021452	0.414334	down
GMW0286*412	135	136	136.052	Benzaldehyde, 2-methoxy-	Aldehyde	135-02-4	1239	sweet, powdery, hawthorn, guaiacol, vanilla, acetophenone, almond	0.174	-	16280.6001651607	15975.5130638119	13084.8916313168	1153.2975477111 64	5766.4877385558 2	1153.2975477111 64	10628.3926890714	9995.87285279828	9848.2659250181 1	1.095464544	0.004149	0.178053	down
KMW0429*337	95	121	182.131	Isobornyl formate	Ester	1200-67-5	1233	camphor, musty, medicinal, woody	-	-	12649.1430159301	12593.3239141234	9462.6753090209 6	3081.66774728392	2824.4337604980 4	2155.5793049316 3	7075.56330902097	7149.09118115232	8218.5029660644 2	1.215907326	0.009938	0.232291	down
XMW0849	107	149	222.198	2-Naphthalenemethanol, 2,3,4,4a,5,6,7,8-octahydro-.alpha.,.alpha.,4a,8-tetramethyl-, [2R-(2.alpha.,4a.beta.,8.bet	Terpenoids	63891-61-2	1606	-	-	-	71658.1621164536	75280.0540788553	70778.5736455061	190008.841900873	191670.251444818	184804.398381586	127213.845788814	128374.790812252	130757.431983394	1.2388318	5.32E-06	2.601928	up

				a.)]-																98			
D264	41	81	138.141	1,6-Octadiene, 3,7-dimethyl-	Terpenoids	2436-90-0	946	citronellol, herbal, citrus, terpenic	-	-	8189.8111201709	7435.431438 41544	4435.2448398742	1599.633464 93527	1279.49019076536	1057.051656524 65	4464.22347985832	4507.72958929439	3369.55598980709	1.192478805	0.040402	0.196215	down
XMW1158	135	93	181.11	Tricyclo[3.3.1.1(3,7)]decane, 2-nitro-	Nitrogen compounds	54564-31-7	1514	-	-	-	175101.554829223	170413.109238035	148748.20843286	62037.9865915524	59818.5653759761	55783.6861433101	108298.901353759	112383.289813231	110867.750692137	1.231548908	0.004217	0.359404	down
XMW1127	56	70	158.131	Formic acid, octyl ester	Ester	112-32-3	1114	fruity, rose, orange, waxy, cucumber	-	-	6653.38099102787	6948.35146203618	8476.3196176148	17076.1304196779	19426.1979116213	15788.1095715333	16411.9590109864	14807.3524656983	15887.4236639406	1.211246889	0.00341	2.368435	up
XMW0973	67	136	151.1	Ketone, methyl 2,4,5-trimethylpyrrol-3-yl	Ketone	19005-95-9	1557	-	-	-	1262.92145617674	1406.62237866208 99	1416.653976562 49	11489.7362951658	12183.3163144529	12665.6821986692	5177.86603527823	4446.2350777587	4569.58213476554	1.23856	0.00812	8.893043	up

XMW0992	134	161	202.172	(2R,8R,8aS)-8,8a-Dimethyl-2-(prop-1-en-2-yl)-1,2,3,7,8,8a-hexahydronaphthalene	Terpenoids	5090-61-9	1511	-	-	-	175484.54622528	174566.252916503	151669.395423462	85437.4537359625	82999.631945191	80401.904950683 9	123329.353700073	129733.170221924	125956.680545044	2151.227437793	0.006881	0.495972	down
XMW0998	141	198	198.119	4-t-Butyl-1-isopropyl-1H-imidazole-2-thiol	Alcohol	61747-36-2	1632	-	-	-	104323.438592273	115438.88578795	119930.719310045	36344.2477711142	45334.8905559035	41115.6062864335	65379.3182372973	69757.9328845138	72741.34509581 71	1.230136265	0.000676	0.361487	down
XMW1081	54	79	119.004	Methanesulfonylacetonitrile	Nitrogen compounds	2274-42-2	1170	-	-	-	6541.26116412353	6775.71971170044	7007.180259826 66	341.093067562866	1951.175676330 57	1705.465337814 33	2999.47988977047	4501.101798645	4578.648777252 21	1.070188892	0.005547	0.196699	down
XMW0925	93	107	150.104	Camphenone, 6-	Ketone	55659-42-2	1095	-	-	-	15806.8833261563	16435.1071327817	15587.3266965635	1906.42524020382	2363.363024383 47	1475.771030090 28	9190.05376174911	8732.82875303635	8595.539761413 42	71.2304	2.62E-06	0.120126	down

																				76093			
XMW0927	96	68	152.084	2,6,6-Trimethyl-2-cyclohexene-1,4-dione	Ketone	1125-21-9	1146.95187165775	musty, woody, sweet, tea, tobacco, leafy	0.025	-	9041.5861783142609	9483.707714294447	8359.395294250556	2631.90316738896	2881.4023247681	2817.930194900 55	6259.44443374641	6035.57679103095	6126.531538330 13	1.23852355	0.001837	0.309888	down
XMW0933	134	119	194.131	Bicyclo[3.1.1]hept-2-en-6-ol, 2,7,7-trimethyl-, acetate, [1S-(1.alpha.,5.alpha.,6.beta.)]-	Ester	50764-55-1	1239	-	-	-	57827.0594250475	55214.9066795337	47823.3432142629	18105.7130452877	17230.1891978756	14013.3446324918	34358.8120020592	34775.7262062218	33325.3654230645	1.222455801	0.002405	0.306774	down
XMW0949	55	57	154.136	1-Decen-3-one	Ketone	56606-79-2	1185	-	-	-	12918.062975647	13775.6746197511	12497.8441558228	4441.86007604982	4118.217687866 23	4011.855466003 42	8202.2158547974	9360.63824786383	8602.719647949 26	1.238166283	0.000658	0.320781	down
D282	81	83	238.193	Citronellyl tiglate	Terpenoids	24717-85-9	1658	rose, fruity, floral, geranium, leafy,	-	-	19996.2780478509	20428.4329897455	23822.241093261	7340.910606 56716	4898.916816406 09	9325.9003656004	13051.7280667721	12914.3592412106	10691.1767043453	1.173	0.001294	0.335669	down

								tea												301243			
D299	121	41	154.136	Ethanol, 2-(3,3-dimethylcyclohexylidene)-, (Z)-	Alcohol	26532-23-0	1225	-	-	-	21819.5895724944	20549.0842452239	16024.9498263549	5412.94525830077	5280.1646451721	4467.75308419798	12683.182512207	12711.1227597045	13239.61636877433	1.222341706	0.012718	0.259632	down
D325	93	121	218.167	(3R,3aR,7S,8aS)-3,6,8,8-Tetramethyl-4,7,8,8a-tetrahydro-1H-3a,7-methanoazulen-2(3H)-one	Terpenoids	288249-25-2	1640	-	-	-	281270.658256313	306041.797650352	313045.505130332	134155.42328148	146752.912733871	140480.651056381	196897.958441627	209200.11227146	206659.177490698	1.236198279	0.001345	0.468024	down
XMW0430	98	70	98.084	2-Piperidinimine	Heterocyclic compound	22780-54-7	1102	-	-	-	6378.47952757264	7748.08525653077	6851.09454147341	15025.313556305	15568.5460897218	13198.7852614136	13193.9143978882	11937.5395002442	14465.0790923767	1.211502194	0.002204	2.087585	up
XMW0441	78	103	102.994	1H-1,2,4-Triazole, 3-chloro-	Heterocyclic compound	6818-99-1	1237	-	-	-	13188.0829296261	11982.6440747066	10353.735638427	4079.04017279042	3751.3238684996	3115.3662763671	7971.15098297	7389.04348095	7100.7782444456	1.2	0.005373	0.308118	down

													4		6		096	681		1820297			
XMW0476	91	193	193.074	Benzoic acid, 2-(acetylamino)-, methyl ester	Ester	2719-08-6	1608	mild, fruity, strawberry	-	-	40171.5101296377	40853.7514404286	39794.64419921 81	93152.8620642063	95856.9112651339	92766.0361232883	62462.9906723616	66099.2637226542	67754.80331445 13	1.240020034	9.78E-05	2.332197	up
XMW0497	139	111	139.993	2,5-Thiophenedicarboxaldehyde	Aldehyde	932-95-6	1220	-	-	-	18849.7730135192	16664.1030603485	14366.993002365	754.3473408 08104	3771.736704040 52	754.3473408081 04	10064.3479640197	9552.91582290648	10094.45435188 29	1.141389423	0.001074	0.105861	down
D317	108	121	208.183	2-Buten-1-ol, 2-ethyl-4-(2,2,3-trimethyl-3-cyclopenten-1-yl)-	Alcohol	28219-61-6	1559	woody, sandalwood, greasy, oily, waxy	-	-	1815.20683557126	2072.02307238765	2158.946771179 17	6034.11721081531	7197.591025390 51	7076.977736938 34	3709.7449499511	4303.3997615966	4603.479073608 31	1.228062691	0.003655	3.35893	up
XMW0	121	92	121.089	Pyridine, 3-propyl-	Heterocyclic	4673-31-8	1027	sweet, musty,	-	-	17350.145936	14971.55143	11268.1456	4200.321667	3439.87524	2681.62675	9493.4540	9473.2956	9041.94763	1.	0.01956	0.236794	down

522					compound			beany, earthy, woody			5386	79271	526793	75507	755854	665278	9677119	5917964	696284	199503383	3		
XMW0253	85	57	170.203	Undecane, 5-methyl-	Hydrocarbons	1632-70-8	1156	-	-	-	9913.2747281 4946	10252.19024 04175	6950.54712 164308	21301.15978 04261	20953.8129 308777	24331.5998 364107	17435.202 9397583	16953.927 8791504	17859.5198 547364	1.189210948	0.000934	2.455618	up
XMW0272	122	106	122.048	4-Pyridinecarboxamide	Heterocyclic compound	1453-82-3	1410	-	-	C02421	79676.488336 8545	78570.63172 93718	65523.0259 348771	30394.16476 61117	27866.2691 486802	26342.8400 125108	44253.463 2275978	45179.648 2506079	43903.5883 663306	1.225043586	0.006538	0.378081	down
XMW0275	69	41	192.151	3,5,9-Undecatrien-2-one, 6,10-dimethyl-	Ketone	141-10-6	1581	sweet, waxy, citrus, floral, balsamic, dry, dusty, powdery, spicy	0.8	-	15640.998932 6179	15659.22585 21736	13488.4609 442145	34840.38296 81411	34915.7549 340834	33352.2592 383436	25247.653 4743663	24868.249 7066661	25914.7678 232433	1.231779849	5.71E-05	2.302108	up

XMW0297	71	43	88.1	1,4-Butanediamine	Amine	110-60-1	901	animalic, rotten, fishy	22	C00134	5213.65791413877	6403.83311849974	4718.07976678465	16177.8390491028	16730.5861117859	16674.798750351	8087.41377201843	8743.46807649231	7812.92961302184	1.221043514	0.000739	3.035292	up
D335	94	41	136.1	Pyrazine, (2-methylpropyl)-	Heterocyclic compound	29460-92-2	1043	green, vegetable, fruity	0.4	-	18716.0007295532	15996.5317153015	12269.612975769	4483.67811132811	4412.6913890686	3833.8993840637 2	10171.0303615112	9814.96167559814	10277.6328531189	1.212564253	0.024596	0.27096	down
XMW0385	161	204	222.198	Carotol	Terpenoids	465-28-1	1594	pleasent, mild	0.008	C09628	1208736.55284128	1178758.05420139	1121274.32039036	271124.46935109	273084.98518947	271329.69030275	270587.652565681	278511.29861573	272049.532050545	1.240102048	0.000811	0.232429	down
XMW0529	93	95	172.102	Bicyclo[2.2.1]heptane, 2-chloro-1,7,7-trimethyl-, (1R-endo)-	Terpenoids	30462-53-4	1165	-	-	-	40117.9528304446	40994.2443536379	28449.1904434205	92393.0861377264	89142.569183991	99068.6589984138	71394.8637489018	68043.3597531742	71790.9087600102	1.2007907	0.000554	2.561161	up

																			68			
XMW0670	94	68	95.048	Acetonitrile, 2,2'-iminobis-	Nitrogen compounds	628-87-5	1171	-	-	-	36872.5202163698	37180.467555115	24288.4210671388	83452.1568771673	81116.9096453253	87752.3215446784	64060.0947724613	62916.1310588383	65528.7005612797	1.18892195	0.002183	2.56577	up
XMW1167	67	109	220.183	(1R,3E,7E,11R)-1,5,5,8-Tetramethyl-12-oxabicyclo[9.1.0]dodeca-3,7-diene	Terpenoids	19888-34-7	1606	-	0.45	-	21216.1991862787	22032.0248011469	20046.17343859 82	55238.4076197494	55894.3446540511	55521.5810449202	37509.8914626454	39740.6987100819	36707.2135529775	1.238082767	7.44E-05	2.633003	up
XMW0703	112	117	228.209	Hexanoic acid, octyl ester	Ester	4887-30-3	1570	-	5	-	2005.78672666935	1999.84597006227	1847.00477050783	5819.69905902106	5749.53378585823	5763.35482940677	4377.15503860479	4077.72745248418	4126.16610635378	1.239486003	2.16E-05	2.9615	up
XMW0745	97	142	184.146	2(3H)-Furanone, 5-hexyldihydro-4-methyl-, trans-	Ketone	147254-33-9	1514	-	-	-	15366.5825436093	14505.0852181393	12439.4146468808	5495.56563970935	5766.214887146	4767.929573852 47	9391.65842858874	10226.8586693112	9521.976473693 82	1.220296	0.005253	0.378854	down

																				268			
XMW0760	59	43	59.048	Guanidine	Nitrogen compounds	113-00-8	1188	-	-	C17349	1554.04323460385	1779.18534115597	1122.03241964711	5107.98247982779	5185.6992533568	4597.342227310009	4084.86458796684	4153.41470634455	3556.5755341796	1.207033982	0.000202	3.342346	up
XMW0539	152	137	152.084	3,4-Dimethoxytoluene	Ether	494-99-5	1233	-	-	-	322209.819406265	294363.870564239	245727.007205108	73417.259796875	70321.8486924439	54767.76177494810	180578.837763839	178805.894667923	183521.203642974	1.22196893	0.007031	0.230206	down
XMW0651	107	106	107.073	Pyridine, 2,3-dimethyl-	Heterocyclic compound	583-61-9	943	coffee, caramel	-	-	51907.3877372277	45305.6603895868	31691.3384602352	10541.1665172728	8512.89846134937	7312.08252563468	28196.1330605312	26352.5629306026	27086.5072221065	1.20630104	0.026561	0.20454	down
D311	81	109	152.12	3-p-Menthen-7-al	Aldehyde	27841-22-1	1169	-	-	-	16866.0470249025	17259.6514504395	11638.306701294	44039.6056807863	43685.2184835207	50087.5932001345	33453.9664323732	33756.8535261232	33659.0368924562	1.204658	0.000409	3.011371	up

																				4			
XMW0574	68	57	142.099	4-Penten-1-ol, propanoate	Ester	30563-30-5	978	-	-	-	9293.37496868871	8265.04105883766	6736.768551940744	2586.77668231194	1903.979978576611	1500.067693176222	5532.45719177231	5236.47645440657	4600.804087310666	1.197115914	0.006816	0.246585	down
XMW0585	84	42	143.058	5-Isoxazolecarboxylic acid, 4,5-dihydro-5-methyl-, methyl ester, (R)-	Heterocyclic compound	64018-42-4	1071	-	-	-	71571.7693677995	80084.4159996356	93367.2807621484	14221.5509987488	16481.5760549011	12284.8606066284	45914.5896574104	43013.2087636115	41315.4663787543	1.231006236	0.00709	0.175444	down
XMW0632	93	77	136.125	Bicyclo[3.1.1]hept-2-ene, 3,6,6-trimethyl-	Terpenoids	4889-83-2	975	-	-	-	534992.044538745	483984.251417225	375935.515081412	135916.04165112	103130.523173886	88821.0210019055	299472.734677544	300682.968160759	295306.110462334	1.207645395	0.011617	0.235045	down
XMW0639	79	91	202.172	1,4-Methanobenzocyclodecene, 1,2,3,4,4a,5,8,9,12,12a-decahydro-	Terpenoids	74708-73-9	1634	-	-	-	596491.157712816	644224.393852456	668767.219868079	219985.533134984	255406.461614717	241869.770616183	386788.99616255	416193.571265817	409691.562179149	1.235439	0.000557	0.375631	down

D1404	82	67	110.11	1-Octyne	Hydrocarbons	629-05-0	784	-	609	-	13679.7582141876	15885.3424588623	10781.36278359 98	4124.30880459597	4564.795967376 73	4622.8867210846	12826.4607250214	12568.6628959198	13296.2585574951	3061.216661781	0.024772	0.329942	down
D1352	77	81	152.047	Benzoic acid, 3-methoxy-	Acid	586-38-9	1413	-	5	-	108641.750742066	107845.442911744	87553.705233644	45537.1320656751	42236.79747961 54	40267.74330407 82	68856.4898950206	72165.2199039318	70158.71442956 66	1.219474659	0.010652	0.421133	down
YZMW0050	44	70	133.11	Diisopropanolamine	Alcohol	110-97-4	1165	-	10	-	3943.02216064457	3625.6330234375	2692.2888757324 2	8925.01001123047	8487.287116699 24	10227.43920361 33	7661.91680541993	7068.72779467772	8270.642616699 23	1.20245715	0.001306	2.693684	up
YZMW0006	65	79	110.073	3-Cyclohexene-1-carboxaldehyde	Aldehyde	100-50-5	958	-	0.015	-	9328.66540351857	7197.28572213734	5746.6381274413 3	1252.85800076291	1444.907590026 83	1266.25922067258 01	4853.83004389947	3721.98607145684	4321.456758056 58	21.2201	0.027486	0.177978	down

YZMW0054	43	73	104.095	Ethanol, 2-[(2-aminoethyl)amino]-	Alcohol	111-41-1	1072	-	2.8	-	41695.9701270151	42786.4101853648	59143.73144165199	1506.3397274902 38	1506.3397274902 38	7531.698637451 19	27040.858568543	26251.0973715825	27333.77973983 81	656951.164997626	0.009243	0.073415	down
YZMW0085	121	137	167.022	Benzoic acid, 3-nitro-	Acid	121-92-6	1583	-	134	-	37387.4025570693	39352.6290192884	34442.9078552257	82684.9760679961	83353.2113850126	77159.56272864 02	57850.6938933124	59900.0955728779	57609.77426892 26	1.2317578541	0.000103	2.187366	up
YZMW0094	142	192	189.994	3,4-dimethyl-2-(methyldisulfanyl)thiophene	Heterocyclic compound	126876-26-4	1500	-	0.005	-	2651.68553552235	2323.91356957994	1865.045558593 66	4976.27379391458	5527.1670513608	4735.519099364 93	4258.39683892798	4591.20850115949	4636.70675912452	1.1990412	0.001026	2.227708	up
YZMW0128	79	149	182.058	2,3-Dimethoxybenzoic acid	Acid	1521-38-6	1414	-	10	-	181647.889347414	161034.49307715	134039.514092531	71299.8191715108	63441.6087325456	60355.549488649	105655.552253419	109162.790762697	106630.460221925	491.20	0.016698	0.409247	down

YZMW0139	41	57	128.12	4-Heptanone, 3-methyl-	Ketone	15726-15-5	928	-	5e-05	-	21608.7709494017	19686.0956110534	13578.95696057 12	7077.758025 08543	6284.15065914915	4543.628825927 72	13499.0588220214	13374.5443662719	13946.43666903 68	6176671.168299856	0.027825	0.326304	down
YZMW0712	153	165	182.058	Benzoic acid, 2,4-dimethoxy-	Acid	91-52-1	1567	-	30	-	956.898710815462	1444.24705062874	1184.67935220341	17961.8600181883	21674.2535705259	22096.4897738035	10084.6950986175	9971.63414294434	9609.920912689 22	1.231986526	0.004202	17.21573	up
CY_023	39	63	168.058	Dibenzofuran	Heterocyclic compound	132-64-9	1515	-	0.12	C07729	5799.43702613816	5258.30975372306	5259.95934767137	2280.87134832757	2148.80269259638	1943.02684790033	4242.53309400932	4077.24921376024	4181.44560926806	1.228734521	0.000432	0.390539	down
w01	57	68	128.12	Cyclooctyl alcohol	Alcohol	696-71-9	1155	-	10	-	8225.25720227049	8625.69631445312	7766.2632003174	3046.06610070804	3024.27743023683	2106.7208759766	6482.21582037355	5421.91307293701	5006.74746572874	1.20	0.000211	0.332169	down

																				9347598			
w15	81	68	142.136	2-Nonen-1-ol	Alcohol	22104-79-6	1105	sweet, fatty, melon, cucumber, vegetable	-	-	3432.54183496093	4687.01881970218	4339.88296685794	8977.62149487315	9989.66619787606	8388.69720678716	7928.91791821295	7541.98345007329	7014.19920300299	1.191376277	0.001411	2.195602	up
BPMW012	63	64	135.032	2(3H)-Benzoxazolone	Ester	59-49-4	1527.64179104478	-	-	-	8249.56628067013	8436.10268457021	7407.5678639831	3348.90492687988	4039.73747384642	4203.31667538451	5390.1165752258	6382.37045700062	5636.87035049431	1.214974936	0.000631	0.481129	down
YZMW0422	53	137	168.079	Phenol, 2-methoxy-4-(methoxymethyl)-	Phenol	5533-03-9	1412	-	2	-	45298.8095712287	45548.836066285	37765.8642849738	17654.735618775	15502.9500599369	15407.8029226688	27123.4452361456	27973.4922219244	26652.6590585942	1.225371226	0.005742	0.377608	down
XMW1	91	92	207.09	N-Benzyloxy-2-carbometh	Heterocyclic	53084-33-6	1581	-	-	-	34903.123705	36322.97722	33969.9832	78945.91388	76317.1451	75820.3036	52579.946	56046.259	56041.4719	1.	1.06E-0	2.196692	up

336				oxyaziridine	compound						8116	48546	736825	52568	865263	0657	4992693	0468768	113787	238158105	5		
XMW1393	109	95	152.157	1-Undecyne	Hydrocarbons	2243-98-3	1095	-	110	-	37323.8045042565	38820.4564062187	37457.38852490166	4443.136167 84656	5086.583435058 47	3293.2395099486 4	19316.2090943599	19470.3234500728	18647.32505368	1.231938282	1.31E-06	0.112877	down
XMW1246	69	55	170.131	2-Butenoic acid, hexyl ester	Ester	19089-92-0	1238	sweet, caramel, green, walnut, oily, radish	-	-	1648.95657452389	2147.24688818355	1623.4804755554	10990.6762796018	11532.5160248411	11596.86073052 96	8258.17445159902	8839.89558270253	7047.9512149352	1.231710647	3.58E-06	6.29558	up
XMW1176	136	91	204.115	Benzeneacetic acid, .alpha.-cyclopentyl-	Acid	3900-93-4	1714	-	-	-	10118.1858253781	11278.6349082028	12001.03143048 06	1691.89738211056	1606.6127237853 6	2007.2601027221 1	5432.09763037097	6186.34770019518	5194.1697593382 6	1.237653483	0.002351	0.158866	down

XMW1218	58	43	172.146	2-Nonanone, 3-(hydroxymethyl)-	Ketone	67801-33-6	1093	fresh, sweet, green, weedy, earthy, herbal	-	-	6912.39475485224	7608.87383874503	9211.83965036	29379.3385559386	29456.944821411	30296.75130458 05	30462.0529024505	31802.4730409087	30878.59288800 04	1.229520289	0.000174	3.755641	up
XMW1251	157	172	172.002	2-Propanone, 1-(5-methyl-3H-1,2-dithiol-3-ylidene)-	Ketone	1005-55-6	1643	-	-	-	26217.4569239472	29564.9025811126	29097.65771087 33	10892.0209824209	11856.11741894 42	10598.64429479 87	16771.9097759992	19986.572025877	18629.81486804 01	1.236119412	0.001472	0.39287	down
XMW1264	163	136	238.193	Acetic acid, 1-methyl-3-(2,6,6-trimethylcyclohex-1-enyl)propyl ester	Ester	86756-12-9	1579	-	-	-	4260.15499609389	3886.49644042984	3299.26363885512	8365.01720162999	8511.6528984073	8403.554366394 36	6115.04238702416	6970.25432891869	6689.649531982 66	1.221672897	0.003039	2.208668	up
D184	55	81	110.037	Hydroquinone	Phenol	123-31-9	919.116883116883	-	5	C00530	19607.9734378051	18557.1899888	11800.06644171 13	5827.787143 0664	5415.25959774779	4083.04048213193	12454.3303466796	12009.1880917968	11195.42567822 26	1.17511163	0.037019	0.306735	down

XMW1464	92	91	164.12	Benzene, (butoxymethyl)-	Ether	588-67-0	1238	floral, rose	-	-	11589.5801198879	10634.8787239987	8559.0930551145	3617.347170 44059	3604.2250659484	3142.888594680 71	7025.91608874494	6567.82973071272	6262.73867504865	131.217504174	0.014676	0.336688	down
XMW1478	68	69	224.178	Geranyl isobutyrate	Terpenoids	2345-26-8	1514	sweet, floral, fruity, green, peach, apricot, rose	0.013	-	490984.838611132	489499.282976	408099.564510976	183787.045170405	180476.819598139	166210.588352717	301695.685120598	318791.85310845	315982.381608267	1.227869657	0.007253	0.382026	down
XMW2374	69	83	140.084	1,3-Cyclohexanedione, 5,5-dimethyl-	Ketone	126-81-8	1176	-	42.9	-	5858.10207849121	5510.86874926765	6302.060509826 73	441.591911798096	2207.959558990 48	2343.712372863 78	5793.03969708257	4213.55898120117	4288.093879394 57	1.007023293	0.012145	0.282568	down
XMW2563	67	95	126.032	Furyl hydroxymethyl ketone	Ketone	17678-19-2	1087	-	1	-	12149.4197326658	11696.5952100218	10786.3496493529	1049.1736950805 48	1049.1736950805 48	1049.1736950805 48	5245.86847540274	5264.01701770008	5342.243658813 34	1.24016	0.001455	0.090884	down

																			5846				
XMW2909	148	119	148.089	3-Isopropylbenzaldehyde	Aldehyde	34246-57-6	1234	-	0.053	-	12847.4786410214	11937.9916282651	9617.37511132794	3557.8123894958	3586.7749025725702	3210.4288213348	7548.11280851733	6636.84261059556	7469.07761738573	1.224259014	0.013035	0.300993	down
XMW0163	81	82	152.12	Furan, 2-hexyl-	Heterocyclic compound	3777-70-6	1095	-	-	-	19939.1658206174	21443.1942890011	20099.3090899045	1762.445118164	270.37244783934597	1351.86223919673	10103.6776494748	10585.7367108152	10092.3856131589	1.163500803	8.01E-06	0.055052	down
KMW0137	57	43	128.157	Nonane	Hydrocarbons	111-84-2	900	linseed, oily, oily, sweaty	12	-	9242.90134313964	9172.24965344237	8156.57172390743	24619.9740641785	26716.9704525452	27464.8535917207	14708.3677407837	14323.6045849609	13932.0661959533	1.23465285	0.000653	2.965626	up
KMW0122	70	41	114.104	Heptanal	Aldehyde	111-71-7	901.304121022431	fresh, aldehydic, fatty, green, herbal, wine,	0.0028	C14390	22118.6980051726	22149.1365433044	15911.5223634642	55414.407938843	56906.1986423952	60220.7908021091	31378.7073768768	33862.2608802032	29630.2447290649	1.2132	0.000256	2.867119	up

								ozonous												38714			
KMW0114	43	71	118.045236	3-mercapto-2-pentanone	Ketone	67633-97-0	902	sulfury, metallic, roasted, onion, horseradish, potato	0.0007	-	8322.39255462645	9381.06873992918	7092.0967201538	21647.880466034	23756.7474611512	24559.5292537842	13659.978802063	13782.3551661377	10186.1051319885	1.219823403	0.000243	2.821641	up
KMW0112	91	106	106.078	o-Xylene	Aromatics	95-47-6	894.211017740429	geranium	7	C07212	5854.21368609619	6005.09364514159	11485.8607103271	2514.09742901612	2621.364172149 65	2893.704308288 57	5415.44856280517	5837.32898315431	5834.743285217 29	1.136315767	0.109772	0.343933	down
NMW0225	104	163	163.1	Acetamide, N-(2-phenylethyl)-	Amine	877-95-2	1512	-	-	C06746	18140.5449439695	17159.3215929261	15522.8858923644	8200.35662451167	8431.101426788 32	6769.826434326 21	11842.4794663087	12870.9695399169	11532.5287475586	1.2109055	0.001073	0.460449	down
KMW0102	56	43	102.104	1-Hexanol	Alcohol	111-27-3	869.208770257388	ethereal, fusel, oily, fruity, alcoholic, sweet,	0.0056	-	42680.3683148803	44843.4178502808	77417.9628762819	8003.249746795 66	11666.2318453064	7766.520833435 06	31305.5444642944	29526.0222983398	27622.0708988342	1.2010	0.053515	0.166338	down

								green												170991			
NMW0230	42	128	128.059	5,6-Dihydro-5-methyluracil	Heterocyclic compound	696-04-8	1524	-	-	C00906	15442.7114205317	14832.6041012263	12883.45805438199	5861.10127053818	5160.810895843 38	5176.9812795409	9269.43082910137	10122.8114769284	10024.89251336 63	1.227183706	0.004314	0.375333	down
NMW0241	55	82	138.091	Pentylenetetrazol	Heterocyclic compound	54-95-5	1560	-	-	C13692	186.0625901367182	930.3129506835 91	1006.7006769409	28399.7755219723	33171.87183251 91	32435.95418456 99	13656.6264755857	17513.367731201	13850.84142480 45	1.188967399	0.0018	44.27896	up
NMW0766	55	56	128.084	2-Propenoic acid, butyl ester	Ester	141-32-2	896.414913957935	-	0.0029	C10921	11279.1191069945	10355.57838031	8794.150625793 42	27479.890441742	29920.36016970 84	30953.38231219 49	15662.8207321777	16837.1275383911	14820.24755105 59	1.227588618	0.000211	2.903614	up
NMW0768	43	56	99.105	Cyclohexylamine	Amine	108-91-8	862.482235907153	-	9.83	C00571	10865.6395113525	13507.6386813965	20005.77423828 13	1999.65115097047	2380.953596344 02	1835.69208148194	8782.32849578861	7633.35515185548	7349.81112710572	1.21	0.042243	0.140073	down

																				8517664			
NMW0772	68	41	68.037	1H-Pyrazole	Heterocyclic compound	288-13-1	887	-	1020	C00481	2894.06688581847	2869.31989233396	1934.57013940428	8370.93894165043	9519.61282806402	9571.60162821965	5115.45896339417	4944.60568778992	4435.31883493042	1.211800416	0.000256	3.56746	up
NMW0788	121	107	136.125	Bicyclo[2.2.1]heptane, 2,2-dimethyl-3-methylene-, (1R)-	Terpenoids	5794-03-6	951	fresh, herbal, woody, fir, camphor	1.86	C06304	141298.607140089	123715.82218340901	84892.6855346058	28396.5433588253	23675.9546728512	18814.9913186948	75023.2615664815	74560.8807588949	73451.4816211843	1.202462529	0.027887	0.202589	down
QWMW0356	122	123	164.131	Pyrazine, 5-butyl-2,3-dimethyl-	Heterocyclic compound	15834-78-3	1226	earthy, sweet	0.77	-	13708.715681549	12323.5489660033	10478.4320306854	3374.74099998473	3205.16267953491	2435.07346128845	7579.54769189452	7822.51542939757	7381.52759878538	1.217839031	0.006252	0.246913	down
NMW0	105	77	182.073	Benzophenone	Ketone	119-61-9	1635	balsamic, rose,	-	C06354	1241275.1570	1355392.115	1410608.73	444210.5934	507101.138	492648.879	797659.49	855182.42	845272.666	1.	0.00121	0.360335	down

270								metallic, powdery, geranium			2621	05597	580157	68699	331484	787785	4939843	3881243	374896	236205145			
NMW0249	81	69	218.203	(3E,7E)-4,8,12-Trimethyltrideca-1,3,7,11-tetraene	Terpenoids	62235-06-7	1580.69129916567	-	-	C20700	42068.4426794453	43589.19772925	39605.601360109	93069.7844693644	93251.2058530313	86700.0429758947	66680.6234986599	67986.859545657	66643.3773972196	1.235123575	0.000231	2.179578	up
GMW0403	69	109	168.079	Benzene, 1,3,5-trimethoxy-	Ether	621-23-8	1413.59223300971	-	-	-	56427.9455446178	55659.3722788096	45341.4198768931	24372.7195283212	18151.7229971915	17939.8170732412	36939.6002302865	36544.9565523691	37504.54315600067	1.200134337	0.003323	0.384074	down
NMW0269	92	91	196.125	Benzene, 1,1'-(1,3-propanediyl)bis-	Aromatics	1081-75-0	1633	-	-	C14581	431512.761502509	468687.673875915	491498.755395201	149829.003369	172240.457100321	164305.450311504	275477.068854091	297673.7481932	294749.992854089	1.236192056	0.001223	0.349483	down

NMW0281	133	105	190.099	3-Butylisobenzofuran-1(3H)-one	Ketone	6066-49-5	1656	herbal, phenol, celery	0.01	C17854	26109.153820678	24757.9123476555	26568.5178325188	7459.93368237283	8740.279561523 21	8779.380357177 49	15912.0663901362	15623.2772385249	20448.3191252436	1.234706947	2.23E-05	0.322585	down
NMW0285	117	129	202.136	Cinnamaldehyde, .alpha.-pentyl-	Aldehyde	122-40-7	1661.37048192771	sweet, floral, oily, fruity, herbal, jasmin	-	C12288	11712.368332275	12933.6011522213	11529.5967563473	2753.793449 4628	3366.904239013 57	3160.426995605 37	5619.76757592755	5211.00482373031	5633.682706909 01	1.236839637	0.000675	0.256558	down
NMW0287	67	79	232.219	1,8,11,14-Heptadecatetraene, (Z,Z,Z)-	Hydrocarbons	10482-53-8	1664	costus	-	C17535	18107.3290566401	20175.1597971186	23082.2136687005	7253.929737 42657	8203.742542602 36	5082.254695922 71	10890.5826800534	9881.30405310032	12148.7677150875	1.192088184	0.002599	0.334719	down
KMW0380	94	124	166.111	Pyrazine, 2-methoxy-3-(2-methylpropyl)-	Heterocyclic compound	24683-00-9	1181	green bell pepper, pea, galbanum	2e-06	-	22651.9901459347	23980.2273888852	23799.4462809141	5062.432361 11444	5557.472884399 36	4264.195151336 62	13778.7024035795	14054.830456909	13647.923666168	1.2345383	5.52E-06	0.211327	down

KMW0344	97	98	144.151	1-Nonanol	Alcohol	143-08-8	1170.641711229 95	fresh, clean, fatty, floral, rose, orange, dusty, wet, oily	0.0053	C14696	10161.1310746458	13534.6582935178	12062.602934387	3743.8608811034	4253.967808349 51	4868.868685912 96	6817.7872064208	9284.17030987531	8324.348808593 59	841.219113327	0.009944	0.359823	down
KMW0390	88	101	172.146	Octanoic acid, ethyl ester	Ester	106-32-1	1196.997319034 85	fruity, banana	0.04	C12292	5431.9069865264	6068.55271701041	5523.585203643 72	2923.31799919125	2853.083014663 66	2378.0448790588	6718.5851591338	6667.19182611075	6651.472143112 08	1.2180600087	0.000397	0.478996	down
KMW0456	55	70	158.167	1-Decanol	Alcohol	112-30-1	1271.528903295 52	fatty, waxy, floral, orange, sweet, watery	0.023	C01633	2732.38112408449	4777.65046008303	3726.770532043 47	1207.37025711061	566.577823699 99	1187.396940551 77	3187.72319134522	4403.53606414794	4762.373234832 77	1.14493282	0.031467	0.26354	down
KMW0477	86	56	86.037	Butyrolactone	Ester	96-48-0	912.043795620 438	creamy, oily, fatty, caramel	20	C01770	5046.95228738402	4803.73057424925	4159.309304977 41	12752.9406777801	12874.837054702 8	14014.540646347	6882.33100987242	7085.71434718323	6543.279177047 73	31.23153	0.00016	2.829575	up

																				8236			
KMW0442	117	91	136.089	3-Phenylpropanol	Alcohol	122-97-4	1233.49540788763	sweet, spicy, cinnamyl, mignonette, hyacinth, balsamic	0.42	-	78306.6019344464	73506.056874327	61795.3786077562	22593.3910622858	21414.3552433467	18627.4231002041	45233.3071744069	45592.9864345845	44616.25108553985	1.225444662	0.006809	0.293225	down
KMW0224	122	91	122.073	Benzene, (methoxymethyl)-	Ether	538-86-3	969	fruity, ethereal, ylang-ylang, green, floral, hyacinth	-	-	7914.08839945967	6923.46218154892	5206.962415710333	1688.19710105892	1254.156846832244	1213.947474548311	4305.49716712939	4290.84718012999	4394.328205078033	1.212354857	0.018603	0.207354	down
KMW0214	79	52	122.048	Acetylpyrazine	Heterocyclic compound	22047-25-2	1023	popcorn, nutty, corn, chip, bread, crust, chocolate, hazelnut, coffee	0.01	-	18079.3999462281	15736.555737976	12220.68156579559	1959.7543614624 04	1959.754361462404	1959.754361462404	10420.742404541	9849.97498846437	9798.771807312022	1.232025097	0.015795	0.127708	down
KMW0708	161	122	222.198	7-epi-a-Eudesmol	Terpenoids	123123-38-6	1658	sweet, woody	-	-	34879.1821528311	38037.695460204	35347.82714648344	17483.8292551264	17554.06943725533	18547.24594799754	26102.6022126458	27172.9973464348	25830.26191747977	1.238	0.001164	0.494946	down

KMW0712	85	128	184.146	2(3H)-Furanone, 5-heptyldihydro-	Ester	104-67-6	1577	fruity, peach, creamy, fatty, lactonic, apricot, ketonic, coconut	0.0021	C08571	7789.91239598105	7415.91645083636	7896.29317272934	32181.3639506831	35245.34287417 57	34152.366875732	22197.3242207027	22265.8473690792	21590.54465832 48	6347021.240152523	0.000906	4.396959	up
NMW0002	125	97	170.094	2-Furaldehyde diethyl acetal	Ether	13529-27-6	1078	fruity, earthy, mushroom	-	C14280	101.1249312622064	623.602157531742	505.6246563110 32	6909.16193481445	6908.6606178436	7015.71225080871	4383.79431819153	4376.95153276062	4607.574641952 49	1.152187918	0.000335	16.93299	up
NMW0016	67	82	142.099	Acetic acid, cyclohexyl ester	Ester	622-45-7	1043	fruity, sweet, musty, ethereal	0.0016	C12297	32610.811735657	28602.8295056765	22021.1052803346	7823.50992486572	6202.137596191 44	1240.427519238288	17862.1149973755	17672.929352356	16693.728076294	1.053884	0.00582	0.18341	down
NMW0055	41	69	154.136	6-Octenal, 3,7-dimethyl-, (R)-	Terpenoids	2385-77-5	1153	fresh, herbal, citrus	0.03	C09848	6928.97329077147	6768.20042895505	7297.353631225 63	2452.42179589843	2591.7076148987	2125.40186840823	4645.175661499	4308.98544073486	5194.475474853 52	51.231	2.83E-05	0.341495	down

NMW0090	151	94	151.136	Amantadine	Amine	768-94-5	1231	-	-	C06818	85146.5614485475	78421.1003248293	66344.9729541933	20131.7451020203	19458.1013930359	15290.8515494842	48197.1732594605	47030.544360016	49357.7734663087	151791.22336739	0.005747	0.238702	down
NMW0092	81	93	154.136	Cyclohexanol, 2-methyl-5-(1-methylethenyl)-, (1.alpha.,2.alpha.,5.beta.)-	Terpenoids	18675-33-7	1226	-	-	-	8051.59192413325	7112.82708447262	5712.960475097 63	1785.39107922362	2459.214191650 37	2094.5275720825	4642.05605792233	5102.43264605709	4497.16724496457	41.20877623	0.013672	0.303636	down
KMW0625	145	84	228.209	Octanoic acid, hexyl ester	Ester	1117-55-1	1581	fruity, green, waxy, berry, apple, estery	0.07	-	6396.62788000495	7867.73396887222	8125.079831298 99	20270.651887573	20255.217259887 7	19519.657932861 1	12565.4836177366	11792.7720609133	12842.7332918698	51.22290378	0.000347	2.681868	up
KMW0286	137	152	152.095	Pyrazine, 2-methoxy-3-(1-methylethyl)-	Heterocyclic compound	25773-40-4	1097	beany, pea, earthy, chocolat	2e-06	-	562183.991225422	581858.481859302	556750.560119495	76512.0039799332	87231.5778662702	59758.2612124316	307993.9589712	311071.573054147	308109.111270196	41.23	1.55E-06	0.13141	down

								e, nutty												3138108			
KMW0278	111	126	126.014	Ethanone, 1-(2-thienyl)-	Ketone	88-15-3	1092	sulfury, nutty, hazelnut, walnut	0.001	-	9376.6323296049 1	10098.7418978269	9639.488536865 04	350.6975796325 56	1753.4878981627 8	350.697579632556	4940.9301151885	5374.80332081594	5435.35358535755	1.158497718	0.000645	0.084317	down
KMW0276	56	55	130.136	1-Octanol	Alcohol	111-87-5	1069.76369495166	intense citrus, rose	0.022	C00756	106786.793971804	112759.948489444	137964.493991092	21877.7004397278	25499.5270043641	18855.6351312866	67796.7702641006	67700.351831117	65158.206915498	1.229192132	0.00762	0.185261	down
KMW0262	55	70	140.120115	1-Nonen-3-one	Ketone	24415-26-7	1076	pungent, mushroom	1e-06	-	81397.5557053244	87827.1960421166	111116.335860232	15878.9293913575	18066.9226008301	13867.1197487183	51375.1629152233	49824.9323557139	47729.0727618418	1.229736663	0.012143	0.170553	down
KMW0	136	68	238.193	Pentanoic acid,	Ester	10402-47-8	1659	rose, fruity,	-	-	10054.275881	9008.036724	12270.8462	3629.548777	3765.74168	4405.67485	6025.0069	7434.4841	6557.76724	1.	0.01654	0.376629	down

653				3,7-dimethyl-2,6-octadienyl ester, (E)-				pineapple			8356	67017	108761	55727	930043	076892	9011215	3403301	237045	210263818	2		
KMW0660	96	85	196.146	2(3H)-Furanone, dihydro-5-(2-octenyl)-, (Z)-	Ester	18679-18-0	1662	sweet, fatty, waxy, dairy, creamy, fruity	0.0054	-	12180.6223162838	12517.7193616939	16388.25288977	2432.18823046869	436.53197777098205	2182.65988885491	5570.85692877181	7350.72413049298	6174.27872521954	1.116616004	0.004927	0.122945	down
D427	166	82	166.136	2-n-Heptylfuran	Heterocyclic compound	3777-71-7	1195	green, fatty, lactonic, oily, roasted, nutty	-	-	3111.5947614898	3231.87257077019	2912.32092976371	17171.6125721432	20194.1298488765	16656.5123613277	11634.9225193174	11126.5976591184	12084.092263519	1.237023777	0.005166	5.836591	up
WMW0104	69	55	142.136	Cyclooctanemethanol	Alcohol	3637-63-6	1073	-	-	-	80343.7845925312	84277.1155095237	104477.888603457	15971.7406782532	18886.6620166931	14482.4120962982	50172.2622297984	50264.9006836557	48028.77615561	1.230383969	0.008705	0.183356	down

D429	120	119	218.167	2-Methyl-6-(p-tolyl)hept-2-en-4-ol	Terpenoids	38142-57-3	1583	-	-	-	9877.76521081575	9737.05735662857	10464.3709861451	20371.9169256596	20520.4263449711	19694.71084283 48	15661.2159803469	16218.7356895144	15022.15233593 77	1.23855187	8.5E-06	2.014251	up
WMW0201	93	79	136.125	Bicyclo[2.2.1]heptane, 7,7-dimethyl-2-methylene-	Terpenoids	471-84-1	950	camphor	-	-	152701.815324094	131868.62064929	91985.36323962 28	31359.5799348445	25512.381998962	21514.10303713 96	84356.4987031239	78912.9401682728	78357.03370828 14	1.203847006	0.028071	0.208166	down
D425	69	41	192.151	3-Cyclohexene-1-carboxaldehyde, 4-(4-methyl-3-pentenyl)-	Aldehyde	37677-14-8	1519	citrus, grassy, aldehydic, floral, marine	-	-	132010.142658322	128998.067809811	110351.68996520 7	55707.7585710437	54330.52644787 49	48089.49295873 91	84581.0571713851	87493.2605926493	88931.223904295	1.221912582	0.00473	0.425807	down
WMW0099	67	82	124.125	Cyclopentene, 1-butyl-	Hydrocarbons	2423-01-0	938	-	-	-	44654.1242377928	40891.722262329	30043.59883496 08	11671.3657651671	11517.084389862	9535.173683166 47	27173.5639209594	27783.6719335326	25334.03692700 19	1.21013024	0.022037	0.283102	down

WMW0014	69	55	152.12	Bicyclo[3.1.1]heptan-3-one, 2,6,6-trimethyl-, (1.alpha.,2.alpha.,5.alpha.)-	Terpenoids	547-60-4	1160	spicy	-	-	14417.2613393555	13145.5502428588	13411.37862005 62	4542.42373391726	5218.619067810 11	4331.618127685 59	9321.23401373294	9510.9127330933	9410.682579864 55	71.230616921	0.000105	0.34394	down
D81	81	94	168.079	2-Furanpropanoic acid, ethyl ester	Ester	10031-90-0	1178	pineapple, fruity, spicy, jammy	-	-	18864.3564106444	19480.2610736082	18524.99013934 32	4702.89526504514	4754.3067678527505	3837.826760070 78	10591.6185321044	11398.4125399169	10868.59068572 99	1.23459424	3.86E-06	0.233781	down
D767	91	131	190.136	Benzenepropanal, 4-(1,1-dimethylethyl)-	Aldehyde	18127-01-0	1521	-	0.1	-	419380.669870845	415121.713986813	367274.49181689 1	189941.271145506	181587.79201367	177658.808123045	279549.690165282	296976.053541258	286081.43850927 5	1.231377459	0.004306	0.45698	down
D446	98	81	224.141	Octanoic acid, 2-furanylmethyl ester	Ester	39252-03-4	1566	sweet, waxy, metallic, fruity, peach	-	-	850.179568145764	1421.04441363531	1921.03954229739	29190.8776431577	34828.92746102 88	34625.28172582 97	15880.8116886748	15351.6169003294	15824.91408892 81	1.2233465	0.002761	23.53027	up

XMW0166	41	69	152.12	3,7-Nonadien-2-one, 8-methyl-, (E)-	Ketone	35408-14-1	1230	-	-	-	6899.64991088862	6507.94970593257	5669.4443109741	2225.33288244627	2463.989803436 26	1604.069423767 08	4520.23194372555	4530.14804064938	4465.107583190 89	381.20018255	0.001089	0.329893	down
XMW0178	105	175	250.193	Acetic acid, (1,2,3,4,5,6,7,8-octahydro-3,8,8-trimethylnaphth-2-yl)methyl ester	Ester	314773-27-8	1748	-	-	-	7384.1638561404	7960.1217467044	10267.8895883792	2449.24326184086	2796.599839233 48	1685.477508300 83	4437.1629694215	4728.49940307626	3799.453406738 44	1.195546707	0.01148	0.270626	down
XMW0189	94	79	152.12	3-Cyclohexene-1-acetaldehyde, .alpha.,4-dimethyl-	Aldehyde	29548-14-9	1225	spicy, herbal	-	-	10096.9239388885	9523.8755509338	7981.52381976315	2810.64577780149	2761.313241455 06	2095.895571105 94	5706.02701985166	5685.71973291013	6199.3938481445	1.21806506	0.004415	0.277797	down
XMW0206	108	135	135.054	1H-Pyrazolo[3,4-d]pyrimidin-4-amine	Amine	2380-63-4	1704	-	-	-	8005.6440681768	8942.87553064019	6561.86532952922	3790.98875930804	3984.537852905 49	3811.657054626 66	4804.4749443973	6073.20903326454	4958.571967407 54	91.203657	0.028239	0.492854	down

D348	68	121	154.136	Cyclobutaneethanol, 1-methyl-2-(1-methylethenyl)-, cis-	Alcohol	30820-22-5	1219	-	-	-	10761.3965030516	10634.9364529723	8453.59648590084	2793.793676605 21	2878.797651275 62	1897.926514724 72	5866.84417663572	6149.01125512693	6020.841096771 21	4711.208814951	0.004276	0.253619	down
XMW0051	107	51	107.037	4-Pyridinecarboxaldehyde	Aldehyde	872-85-5	1025	fruity	-	-	15011.3175579223	12833.8780549926	10186.8016366271	3722.662507965 06	3431.676285430 89	686.335257086178	7948.05611679072	8273.69194268796	8385.353945846 49	1.046271641	0.005805	0.20616	down
XMW0114	77	107	107.037	Benzene, nitroso-	Nitrogen compounds	586-96-9	1019	-	0.15	C06876	10685.4675750122	8589.14845404059	6604.749952575 72	2896.824171752 94	1817.142315734 84	2222.433832397 43	6074.36727569582	5360.39893579 1	5730.542887939 45	1.185941125	0.026519	0.268028	down
QWMW1138	77	63	106.045	Ethanol, 2-(ethylthio)-	Alcohol	110-77-0	931	meaty	0.01	-	175300.574491149	163219.942011108	107516.949706847	48871.224865203 7	46185.153898925 6	37265.802757690 3	105356.158086974	104159.279973815	101343.060933135	1.1897	0.034529	0.296662	down

GM W0 200	70	97	112.052	Sorbic Acid	Acid	110-44-1	1068.79 69924812	-	135	-	79687. 085596 5595	83422. 83911 5847	10250 1.218 62158 4	16101. 73952 4292	18992 .9296 64978 1	12560 .6560 19042 9	4954 2.221 2581 797	4744 3.407 0289 315	44873 .8968 87696 1	833171.223758047	0.00 637 3	0.1794 18	down
QW M W1 114	79	69	224.178	Neryl butyrate	Ester	999-40-6	1517	green, waxy, oily, fatty, sweet, leafy, orange	-	-	448457 .04328 5635	43394 4.8388 39102	37401 5.282 78856 7	17759 9.5156 10104	17158 1.008 23901	16518 5.892 36864 9	2822 58.99 3041 987	2989 88.39 3024 408	28790 1.817 20360 6	1.228829945	0.00 724 1	0.4093 91	down
QW M W0 712	133	134	180.079	Benzoic acid, 2-methoxy-, ethyl ester	Ester	7335-26-4	1412	sweet, floral, fruity, raspberry, grape, ylang-ylang	-	-	400921 .69015 2321	39710 0.6505 78103	32603 7.894 35862 5	17116 3.7897 57559	15972 5.116 77953 2	12061 1.521 52587 1	2638 57.96 4890 366	2671 37.29 7943 1	26313 8.741 42015 1	1.193824501	0.00 287 9	0.4016 69	down
QW M W0 729	82	67	170.094	cis-3-Hexenylpyruvate	Ester	68133-76-6	1181	green, spicy, caramel, cucumbe	-	-	6195.6 498704 8342	6349.3 41702 63675	6535. 46345 31860 7	1893.1 75797 69896	1891. 79894 41223 1	2096. 41629 33349 6	4132. 0488 9074 709	4008. 1954 4342 042	3919. 38671 35009 8	1.23	1.01 E-0 5	0.3082 42	down

								r, melon, vegetable												9479027			
QWMW0741	132	151	207.126	Benzoic acid, 2-(methylamino)-, 2-methylpropyl ester	Ester	65505-24-0	1638	sweet, floral, fruity, citrus, grape, grapefruit	-	-	51018.8500472961	54950.5372535333	55240.8579877862	20756.7354982888	23310.8209085668	21839.7271590552	34249.6562300375	35194.661909908	35619.1723348347	1.237541602	0.000211	0.408828	down
QWMW0879	173	188	188.12	5-Methyl-2-phenyl-2-hexenal	Aldehyde	21834-92-4	1493	cocoa	-	-	11712.0539254756	13324.8433144526	12492.3566745602	25444.2817077323	27328.2863111256	25751.2862256458	25781.7497840566	26148.8041603996	24277.9597246389	1.228931533	7.42E-05	2.092337	up
QWMW0901	41	84	198.162	2-Oxepanone, 7-hexyl-	Ketone	16429-21-3	1635	peach, apricot, milky, fatty	-	-	418787.560760814	457465.056858341	471519.31679755	156053.052169415	178772.79117686	170571.319650981	275385.348685147	293677.478228358	291381.97632101	1.236103827	0.000898	0.374987	down
WAM	41	81	180.151	2,4-Dodecadienal, (E,E)-	Aldehyde	21662-16-8	1524	grapefruit,	-	-	93241.231519	92480.04829	80819.5326	39992.30597	38485.7857	36990.8288	63651.395	65921.756	64753.4621	1.	0.00469	0.433213	down

W0913								orange, fatty, citrus			1016	83375	528301	46083	95043	488151	3944691	3098128	558822	230900448	8		
WAMW1043	82	57	226.23	Pentadecanal-	Aldehyde	2765-11-9	1715	fresh, waxy	1	C01948	8460.18900091533	8494.14406689434	10243.9719481198	1732.70469802852	2461.855462890 57	346.5409396057 04	4833.92681292713	5203.10324414051	4277.7487845458	1.065871708	0.00092	0.166963	down
WAMW1065	91	93	136.125	Bicyclo[3.1.0]hex-2-ene, 2-methyl-5-(1-methylethyl)-	Terpenoids	2867-05-2	929	woody, green, herbal	-	-	315654.449731078	294871.752890258	196193.945327575	92232.2087552183	84548.5452672728	68979.8987937621	192581.458575072	190105.524259643	186215.091443695	1.188668262	0.033044	0.304642	down
WAMW1462	92	107	107.073	Pyridine, 3-ethyl-	Heterocyclic compound	536-78-7	961	tobacco, oakmoss, leathery	-	-	17319.5651011959	16643.9387443845	11544.9133063658	3887.6929865722	3525.1491308593 2	2773.2176836852 6	11208.7446337583	9258.52323541244	10531.89242364 48	1.210263793	0.020383	0.223828	down

QWMW1287	94	67	140.12	Non-8-enal	Aldehyde	39770-04-2	1094	smoky, plastic	0.0002	-	21947.4387081753	22092.2357477718	21671.5735819698	2670.24740795894	3606.03435314937	2124.93232757561	11380.3691760251	12050.6628559721	11394.6056128843	1.227082393	0.000207	0.12785	down
QWMW1290	71	55	146.077	Hexanethioic acid, S-methyl ester	Ester	2432-77-1	1083	cabbage, rubbery	0.0003	-	3474.73496441646	1159.00489776608	1324.73639486691	6377.37131903065	13421.4212678526	6490.58006074513	6925.0076150054	7405.19539892566	6079.25864422602	1.100592409	0.089114	4.412097	up
WAMW0570	119	91	191.131	Diethyltoluamide	Amine	134-62-3	1582	-	-	C10935	26411.6751815195	26882.1610570074	25279.1198153085	58853.2390532246	61106.7443688982	56025.3742915055	42104.0138831798	43668.2753806163	43137.1313067638	1.237233919	0.000858	2.23977	up
WAMW0654	109	78	138.068	Benzene, 1,3-dimethoxy-	Ether	151-10-0	1168	acid, fruity, nutmeg, neroli	0.0214	-	3604.33914569094	3813.02417477422	2573.97439376834	9339.30962857064	8997.1696334229	10054.5968803406	7248.27858155826	6964.50202604679	7416.67334561163	1.20493334	0.000305	2.841569	up

																				72			
KMW0226*017	68	93	136.125	Limonene	Terpenoids	138-86-3	1030	citrus, herbal, terpene, camphor	0.01	C06078	48462.9633620151	41052.3093520206	32331.68576837l7	11871.9174226074	9869.132036285 45	8579.829633956 92	26241.7432005769	26082.2092056886	25181.420923187 4	1.208908914	0.019122	0.248844	down
NMW0015*017	68	93	136.125	Cyclohexene, 1-methyl-4-(1-methylethenyl)-, (S)-	Terpenoids	5989-54-8	1030.90128755365	terpene, pine, herbal, peppery	0.034	C00521	48462.9633620151	41052.3093520206	32331.685768371 7	11871.9174226074	9869.132036285 45	8579.829633956 92	26241.7432005769	26082.2092056886	25181.420923187 4	1.208908914	0.019122	0.248844	down
KMW0217*017	68	93	136.125	D-Limonene	Terpenoids	5989-27-5	1031.27001067236	citrus	0.034	C06099	48462.9633620151	41052.3093520206	32331.685768371 7	11871.9174226074	9869.132036285 45	8579.829633956 92	26241.7432005769	26082.2092056886	25181.420923187 4	1.208908914	0.019122	0.248844	down
D1049*095	71	81	156.151	Cyclohexanol, 5-methyl-2-(1-methylethyl)-, (1.alpha.,2.alpha.,5.beta.)-	Terpenoids	3623-51-6	1161	-	0.4	-	65893.998031876	71580.5728064581	70657.245008759	12024.690390405 36	12024.690390405 36	12024.690390405 36	64014.7651022954	60123.4519520268	61429.506632019 5	1.24138	0.000942	0.173323	down

NMW0789*218	105	120	120.094	Benzene, 1-ethyl-3-methyl-	Aromatics	620-14-4	957	-	0.088	C14522	12740.2891000211	10360.7668805845	7497.577710845 83	1240.29215329588	1240.292153295 88	1240.292153295 88	6493.36911451711	6371.21609757985	6201.4607664794	38981.225785032	0.027444	0.121603	down
XMW0301*218	105	120	120.094	Mesitylene	Aromatics	108-67-8	972	-	0.83	C14508	14896.3236640011	13397.4172313229	10634.4999048459	1626.20000357662	1626.200003576 62	1626.200003576 62	8843.62953283672	8131.0000178831	8561.143925750 54	1.23435539	0.011879	0.125323	down
KMW0148*011	93	91	136.125	.alpha.-Pinene	Terpenoids	80-56-8	936.358894105373	fresh, camphor, sweet, pine, earthy, woody	0.274	-	800422.055898131	744229.399208525	493300.796847228	232768.349228149	209861.294824462	172751.408988113	493071.907169844	479857.114023376	472514.67356668	1.18783928	0.033749	0.30196	down
WMW0023*011	93	91	136.125	(1R)-2,6,6-Trimethylbicyclo[3.1.1]hept-2-ene	Terpenoids	7785-70-8	936.472424557752	harsh, terpene, aromatic, minty	0.0053	C06306	800422.055898131	744229.399208525	493300.796847228	232768.349228149	209861.294824462	172751.408988113	493071.907169844	479857.114023376	472514.67356668	91.1878	0.033749	0.30196	down

NMW0302*459	69	107	222.198	2,6,10-Dodecatrien-1-ol, 3,7,11-trimethyl-, (Z,E)-	Terpenoids	3790-71-4	1697	-	-	C03220	5335.20891192646	5035.56424060082	4505.32572045917	2480.94257971199	2615.21044091805	2216.87964373788	3392.11154095473	4035.67044458022	4150.41604638687	392891.2153	0.003034	0.491596	down
NMW0261*318	127	115	173.048	Naphthalene, 1-nitro-	Nitrogen compounds	86-57-7	1612	-	-	C14040	161307.696251872	176079.883867226	184446.179364052	53869.3890836425	63406.794140038	61299.9429431083	100575.399770068	109260.990817858	109372.00748018	136581.2348	0.000999	0.342209	down
D293*137	93	119	204.188	Cyclohexene, 4-[(1E)-1,5-dimethyl-1,4-hexadien-1-yl]-1-methyl-	Terpenoids	25532-79-0	1512	-	-	-	798413.262021469	787644.091966049	666109.812415515	312413.274434564	302920.58750268	282953.520335932	497461.537589348	526587.123727041	509735.035969716	782581.2277	0.006856	0.398855	down
XMW0975*13	93	119	204.188	Cyclohexene, 4-(1,5-dimethyl-1,4-hexadienyl)-1-meth	Terpenoids	17627-44-0	1515	balsamic, spicy, floral	-	-	798413.262021469	787644.091966049	666109.812415515	312413.274434564	302920.58750268	282953.520335932	497461.537589348	526587.123727041	509735.035969716	059191.22	0.006856	0.398855	down

7				yl-																7			
KMW0123*199	41	68	112.089	4-Heptenal, (Z)-	Aldehyde	6728-31-0	900	oily, fatty, green, dairy, milky, creamy	2.5e-05	-	4315.64540960693	3923.95670809936	3456.07794714354	13551.2488166504	12974.2455733643	14282.5470191651	7481.32861962891	6234.18448602295	6686.2235975647	7059191.234416059	8.51E-05	3.489155	up
D1362*181	55	41	100.052	4-Pentenoic acid	Acid	591-80-0	882.071901608325	-	-	-	19905.892184204	21324.6982518005	36425.86051412 97	4088.58513793946	5565.31051229859	3263.27225018309	14085.7504959716	12390.0418035278	12364.3120760193	1.195886222	0.052987	0.166337	down
D1346*198	69	56	112.089	Cyclohexanone, 2-methyl-	Ketone	583-60-8	953	-	0.83	-	3806.34166540525	3493.85394396968	7249.751690338 11	13209.4958898619	11877.6597295226	13557.9709316252	7328.76979844665	7872.18545567318	7234.815936309 81	1.1320091	0.011701	2.656032	up
KMW0	112	69	154.136	Cyclohexanone,	Terpenoids	89-80-5	1154	minty	-	-	81952.177339	85370.59263	55835.4316	183925.5798	181735.192	198027.668	146754.63	141377.65	146374.444	871.	0.00151	2.525959	up

313*112				5-methyl-2-(1-methylethyl)-, trans-							9051	34843	630251	8672	689088	99141	1098328	2801819	99762	189455375	8		
QWMW1015*112	112	69	154.136	L-Menthone	Terpenoids	14073-97-3	1158.98395721925	menthol, pepperminty, herbal, camphor	0.17	C00843	81952.1773399051	85370.5926334843	55835.4316630251	183925.57988672	181735.192689088	198027.66899141	146754.631098328	141377.652801819	146374.44499762	1.189455375	0.001518	2.525959	up
NMW0062*112	112	69	154.136	Cyclohexanone, 5-methyl-2-(1-methylethyl)-, cis-	Terpenoids	491-07-6	1164	minty, cool, pepperminty, sweet	-	-	81952.1773399051	85370.5926334843	55835.4316630251	183925.57988672	181735.192689088	198027.66899141	146754.631098328	141377.652801819	146374.44499762	1.189455375	0.001518	2.525959	up
KMW0143*211	45	55	116.12	2-Heptanol	Alcohol	543-49-7	900.361197110423	fruity, mouldy, musty, mushroom	0.1	-	2739.51089807127	2762.64667807007	2653.09248400878	6960.46241250613	7094.1097773743	7852.30238706973	4562.07834967041	4295.17425628662	3955.81262411499	1.235984325	0.003273	2.68623	up

KMW0385*093	93	121	154.136	Cyclohexanol, 2-methyl-5-(1-methylethenyl)-	Terpenoids	619-01-2	1196	minty, spice	-	C18017	2495.7618508148	2439.600777786251	2088.849685729 95	6725.57979083238	7800.96759994485	6182.26023985279	4781.39365234367	5554.52130232231	4865.3523726195	1.225601724	0.007362	2.948204	up
KMW0389*092	95	110	154.136	endo-Borneol	Terpenoids	507-70-0	1170.415778251 6	pine, woody, camphor, balsamic	0.18	C01411	188148.340954591	196957.63712964	132272.609931214	420459.531877504	413035.676212282	450059.618242495	334984.835125429	324374.154021791	330198.789915255	1.19377539	0.001308	2.480881	up
XMW0389*382	111	123	222.198	Caryophyllenyl alcohol	Terpenoids	913176-41-7	1574	-	-	-	1894.00623474118	2617.92797650146	2779.8048170166	23474.5361446531	28240.4720574949	27912.5851895749	13398.8031282958	12900.4769909056	12030.2319331054	1.230254972	0.003211	10.92025	up
D441*142	136	121	238.193	Butanoic acid, 3-methyl-, 3,7-dimethyl-2,6-octadienyl ester, (Z)-	Ester	3915-83-1	1582	sweaty, floral, rose, bergamot, citrus, fruity	-	-	7781.81138055458	9184.51130798377	7919.70201367217	18795.6763169871	17421.7143131723	17231.3859595954	12273.4917277227	12662.0143253178	11847.2723691717	1.22288496	0.0147	2.147743	up

7

XMW0556*064	189	161	204.188	(2S,4aR,8aR)-4a,8-Dimethyl-2-(prop-1-en-2-yl)-1,2,3,4,4a,5,6,8a-octahydronaphthalene	Terpenoids	123123-37-5	1517	-	-	-	566681.342471687	548975.284249031	469131.018474371	225457.92882654	220209.992075075	212681.360136721	355074.379186161	372384.375910649	362740.796031622	1.228135316	0.008529	0.415418	down
KMW0377*042	82	57	184.146	cis-3-Hexenyl-.alpha.-methylbutyrate	Ester	53398-85-9	1234	fresh, green, apple, sweet, fruity, pear	-	-	1345.16392333981	1610.00808883663	269.0327846679 62	6500.40905203238	6753.66899667 35	6659.293238418 46	4623.15826757805	4133.24018054192	4053.240807769 71	1.085721306	0.004332	6.176212	up
XMW1401*042	82	67	184.146	n-Valeric acid cis-3-hexenyl ester	Ester	35852-46-1	1237	green, fruity, apple, pear, kiwi, unripe, banana, tropical	0.06	-	1345.16392333981	1610.00808883663	269.0327846679 62	6500.40905203238	6753.66899667 35	6659.293238418 46	4623.15826757805	4133.24018054192	4053.240807769 71	1.085721306	0.004332	6.176212	up
KMW0366*313	67	82	170.131	Butanoic acid, 3-hexenyl ester, (Z)-	Ester	16491-36-4	1187	fresh, green, apple, fruity, wine, metallic, buttery	20	-	6984.82780780033	6638.72054156496	6994.663201843 29	2511.92282153 32	2285.124359863 27	2948.458842224 13	5555.31633880618	5138.43358398438	5054.933760772 71	1.226375	0.00185	0.375663	down

																				464			
XMW1351*361	136	121	204.188	(3aS,8aS)-6,8a-Dimethyl-3-(propan-2-ylidene)-1,2,3,3a,4,5,8,8a-octahydroazulene	Terpenoids	395070-76-5	1532	-	-	-	43036.4350910642	42748.9412239988	36546.3212392574	14801.7596207274	14642.5578370361	13730.139649536	26340.609210022	28261.2364161984	27338.9459044186	1.232718734	0.005435	0.352929	down
D376*447	150	135	150.104	(Z)-2,6-Dimethylocta-2,5,7-trien-4-one	Ketone	33746-71-3	1231	-	-	-	118262.37671388	111366.516603086	93608.7867880688	34385.2358811334	31939.1217118982	27131.1968817286	67487.6549403826	68061.8603887162	67319.9895203081	1.223284162	0.005717	0.289123	down
XMW0202*261	69	41	138.141	cis-2,6-Dimethyl-2,6-octadiene	Hydrocarbons	2492-22-0	997	-	-	-	40120.1400521238	41377.4183155515	26950.9127198484	12442.2581413268	9504.22599667354	10025.7920770873	25619.7656520384	29856.5257326047	22763.4119666746	1.198856364	0.027471	0.294815	down
D259*254	51	93	136.089	Phenol, 2,3,6-trimethyl-	Phenol	2416-94-6	1240	-	-	-	8851.89920005773	8174.38603442357	6896.33458779885	2769.08507675162	2343.25752334588	2230.51027613824	5556.19422334274	5212.83944894396	5098.24167135607	1.2215	0.006927	0.306942	down

XMW0192*015	79	121	136.125	(+)-4-Carene	Terpenoids	29050-33-7	1009	-	-	-	11487.4166761473	12633.6235126341	9801.056311706 46	3863.900402 40473	2889.45682714842	3127.959033203 09	8238.74503100576	7144.36733544915	7825.445639160 07	290351.220758112	0.005481	0.291294	down
XMW0006*015	79	121	136.125	(+)-3-Carene	Terpenoids	498-15-7	1010	sweet	0.044	C11382	11487.4166761473	12633.6235126341	9801.056311706 46	3863.900402 40473	2889.45682714842	3127.959033203 09	8238.74503100576	7144.36733544915	7825.445639160 07	1.220758112	0.005481	0.291294	down
KMW0220*015	79	121	136.125	3-Carene	Terpenoids	13466-78-9	1012.4597207304	citrus, terpenic, herbal, pine, solvent, resinous, phenol, cypress, medicinal, woody	9.3	-	11487.4166761473	12633.6235126341	9801.056311706 46	3863.900402 40473	2889.45682714842	3127.959033203 09	8238.74503100576	7144.36733544915	7825.445639160 07	1.220758112	0.005481	0.291294	down
KMW0290*02	82	71	152.12	Hotrienol	Alcohol	20053-88-7	1106	hyacinth	0.11	-	4761.94916809083	6646.09709020999	5232.977543701 19	12977.3273486329	14382.0876923829	9551.69933734138	11537.7555530396	9681.14264636237	9920.33029113775	1.14	0.029229	2.21808	up

4																							
KMW0193*119	41	93	136.125	.beta.-Pinene	Terpenoids	127-91-3	979.717720857292	dry, woody, resinous, pine, hay, green	0.14	C09882	39601.1848816518	36995.6447611074	27771.86976965 26	8995.549897 73534	7455.0697960813 3	5637.9246855467 1	21717.5649625 238	22229.9415914 301	20507.932061156 695	72507941.208432098	0.012136	0.21164	down
KMW0235*018	93	91	136.125	1,3,6-Octatriene, 3,7-dimethyl-, (Z)-	Terpenoids	3338-55-4	1038	warm, floral, herbal, flowery, sweet	0.034	C22910	46268.845510147	40248.3136492005	30387.746502655	11768.8962056579	9412.4851000365 5	8245.0859780730 9	25212.1105211 486	24858.3189111 785	23780.643292907 6	1.2045282721	0.020125	0.251713	down
KMW0252*018	93	91	136.125	1,3,7-Octatriene, 3,7-dimethyl-	Terpenoids	502-99-8	1048	fruity, floral, wet, cloth	-	-	46268.845510147	40248.3136492005	30387.746502655	11768.8962056579	9412.4851000365 5	8245.0859780730 9	25212.1105211 486	24858.3189111 785	23780.643292907 6	1.2045282	0.020125	0.251713	down
WM	93	91	136.125	trans-.beta.-Ocimene	Terpenoids	3779-61-1	1049	sweet, herbal	0.034	-	46268.845510	40248.31364	30387.7465	11768.89620	9412.48510	8245.08597	25212.110	24858.318	23780.6432	721.	0.02012	0.251713	down

W0040*018											147	92005	02655	56579	003655	807309	5211486	9111785	929076	204528272	5		
KMW0345*032	70	41	138.104	2,6-Nonadienal, (E,Z)-	Aldehyde	557-48-2	1154.74919957311	cucumber, green	1e-05	C08499	21064.7802434082	20542.8595093994	19894.8598374634	6603.0160802918	6977.31862081915	6186.33728698738	14233.3704739381	15281.0379852295	14885.2997673341	1.237342588	1.5E-05	0.321396	down
KMW0070*006	91	106	106.078	Benzene, 1,3-dimethyl-	Aromatics	108-38-3	870.582662245381	plastic	1	C07208	13085.7395057373	14006.2112203369	24086.5492182617	4879.75911813355	4335.31010388183	4726.98948266602	9381.38698034668	11329.5919867554	12902.8789102478	1.1855258	0.07169	0.27242	down
XMW0307*006	91	106	106.078	p-Xylene	Aromatics	106-42-3	871.103742302226	strong, sweetish	1	C06756	13085.7395057373	14006.2112203369	24086.5492182617	4879.75911813355	4335.31010388183	4726.98948266602	9381.38698034668	11329.5919867554	12902.8789102478	1.1855258	0.07169	0.27242	down
KMW0460*28	93	69	154.136	Geraniol	Terpenoids	106-24-1	1255.60949298813	sweet, floral, fruity, rose,	0.0066	C01500	14414.4097798305	13223.7075906063	10600.237887695	5120.95969131459	4907.68927313221	4221.55241308583	8523.43434260538	8878.05979618814	8737.65537341287	1.20	0.015156	0.372668	down

5								waxy, citrus												8794528			
NMW0104*285	93	69	154.136	2,6-Octadien-1-ol, 3,7-dimethyl-	Terpenoids	624-15-7	1260	-	-	-	14414.4097798305	13223.7075906063	10600.237887695	5120.95969131459	4907.689273132 21	4221.552413085 83	8523.43434260538	8878.05979618814	8737.655373412 87	1.208794528	0.015156	0.372668	down
D327*359	187	159	202.172	(4R,4aR)-4,4a-Dimethyl-6-(prop-1-en-2-yl)-1,2,3,4,4a,7-hexahydronaphthalene	Terpenoids	28908-26-1	1537	-	-	-	20459.8230146483	23199.935709778	19529.3853854982	9705.432963 74512	8970.194004028 36	8132.567005615 24	16123.6810369873	15055.8082293701	15384.443326172	1.225129743	0.003327	0.424253	down
D397*067	109	82	226.193	11-Dodecen-1-yl acetate	Ester	35153-10-7	1604	-	-	-	49514.9297277819	48633.377858519	43532.5192370592	108434.042118527	111817.716706905	106167.069566158	82525.7311470922	84417.0341856662	81505.61604833 74	1.234955051	1.8E-05	2.303903	up
XMW0186	95	43	124.089	3,5-Octadien-2-one, (E,E)-	Ketone	30086-02-3	1073	fruity, green, grassy	0.0005	-	35874.9083925012	37055.984670318	35410.008375396	1010.797120 42844	5053.985602 1422	1010.797120 4284	19069.7013900	18777.4092108	19231.203988128	1.1	0.000509	0.065308	down

*139													2	8	4	48	753	761	3	70530193			
KMW0343*263	55	70	140.12	2-Nonenal	Aldehyde	2463-53-8	1161	fatty, green, waxy, cucumber, melon	0.0001	-	18230.515453064	19083.3957853393	17292.5926511231	6179.70374179081 05	6589.547097900 43	5728.456563476 62	14313.5585128174	13467.3359269409	13518.2281118774	1.236263094	0.000308	0.338745	down
KMW0338*263	55	70	140.12	2-Nonenal, (E)-	Aldehyde	18829-56-6	1161.20597652081	fatty, green, cucumber, aldehydic, citrus	8e-05	-	18230.515453064	19083.3957853393	17292.5926511231	6179.70374179081 05	6589.547097900 43	5728.456563476 62	14313.5585128174	13467.3359269409	13518.2281118774	1.236263094	0.000308	0.338745	down
D272*145	70	85	172.146	Butanoic acid, 3-methyl-, 2-methylbutyl ester	Ester	2445-77-4	1107	herbal, fruity, earthy, cheese, apple, green	-	-	9098.47357666019	8694.37298919683	7646.04262811282	15684.2280886537	20096.3453300783	15593.3526766968	15538.2391706544	15478.2283851319	15793.0882939759	1.19540691	0.021334	2.019504	up
D289*1	70	85	172.146	n-Amyl isovalerate	Ester	25415-62-7	1110	apple, fresh	12	-	9098.4735766	8694.372989	7646.04262	15684.22808	20096.3453	15593.3526	15538.239	15478.228	15793.0882	1.	0.02133	2.019504	up

45								fruit			6019	19683	811282	86537	300783	766968	1706544	3851319	939759	19540691	4		
KMW0157*012	41	83	112.089	2-Heptenal, (E)-	Aldehyde	18829-55-5	958	pungent, green, vegetable, fresh, fatty	0.013	-	3628.85362329103	4879.37319482424	3380.17169781496	9593.44851898187	9027.25895294181	9664.39399650568	6185.27920471189	6326.87885449215	6529.18041366574	1.194180658	0.002558	2.379219	up
KMW0452*080	133	105	148.089	Benzaldehyde, 4-(1-methylethyl)-	Terpenoids	122-03-2	1246.38619201726	spicy, cumin, green, herbal	0.4	C06577	24889.7815196527	25172.8040430598	19634.930598022	8072.25610342393	7171.7555282286	6715.53960884082	16110.9029293057	15079.0560270382	14500.3088358456	1.222863139	0.009832	0.315069	down
XMW0926*080	133	105	148.089	Ethanone, 1-(2,4-dimethylphenyl)-	Ketone	89-74-7	1253	floral, woody, sweet, mimosa, minty	-	-	24889.7815196527	25172.8040430598	19634.930598022	8072.25610342393	7171.7555282286	6715.53960884082	16110.9029293057	15079.0560270382	14500.3088358456	1.222863139	0.009832	0.315069	down
KM	103	57	186.162	Butanoic	Ester	10032-	1236	green,	0.022	-	22773.	21872.	18276	6472.5	6785.	5088.	1309	1326	13074	1	0.00	0.2915	down

W0424*342				acid, 2-methyl-, hexyl ester		15-2		waxy, fruity, apple, spicy, tropical			8852793725	4943322443	.2561313472	442756041	50315203843	81099017321	9.1921672512	2.3991186672	.2670603329	.2192787511	3917	78	
D219*412	135	136	136.052	Benzaldehyde, 4-methoxy-	Aldehyde	123-11-5	1259.75148568341	sweet, powdery, mimosa, floral, hawthorn, balsamic	0.0002	C10761	16280.6001651607	15975.5130638119	13084.8916313168	1153.2975477111 64	5766.4877385558 2	1153.2975477111 164	10628.3926890714	9995.87285279828	9848.26592501811	.0954645441	0.004149	0.178053	down
KMW0449*337	95	121	182.131	Bicyclo[2.2.1]heptan-2-ol, 1,7,7-trimethyl-, formate, endo-	Ester	7492-41-3	1226	green, earthy, herbal, balsamic, pine	-	-	12649.1430159301	12593.3239141234	9462.675309020 96	3081.66774728392	2824.433760498 04	2155.5793049316 3	7075.56330902097	7149.09118115232	8218.50296606442	.215907326	0.009938	0.232291	down
